# A method to generate the surface cell layer of the 3D virtual shoot apex from apical initials

**DOI:** 10.1186/s13007-017-0262-7

**Published:** 2017-12-11

**Authors:** Krzysztof Kucypera, Marcin Lipowczan, Anna Piekarska-Stachowiak, Jerzy Nakielski

**Affiliations:** Department of Biophysics and Morphogenesis of Plants, University of Silesia, Katowice, Poland

**Keywords:** Apical initials, Cell growth and division, Computer simulation, Shoot apex, Surface cell layer, Tensor growth field

## Abstract

**Background:**

The development of cell pattern in the surface cell layer of the shoot apex can be investigated in vivo by use of a time-lapse confocal images, showing naked meristem in 3D in successive times. However, how this layer is originated from apical initials and develops as a result of growth and divisions of their descendants, remains unknown. This is an open area for computer modelling. A method to generate the surface cell layer is presented on the example of the 3D paraboloidal shoot apical dome. In the used model the layer originates from three apical initials that meet at the dome summit and develops through growth and cell divisions under the isotropic surface growth, defined by the growth tensor. The cells, which are described by polyhedrons, divide anticlinally with the smallest division plane that passes depending on the used mode through the cell center, or the point found randomly near this center. The formation of the surface cell pattern is described with the attention being paid to activity of the apical initials and fates of their descendants.

**Results:**

The computer generated surface layer that included about 350 cells required about 1200 divisions of the apical initials and their derivatives. The derivatives were arranged into three more or less equal clonal sectors composed of cellular clones at different age. Each apical initial renewed itself 7–8 times to produce the sector. In the shape and location and the cellular clones the following divisions of the initial were manifested. The application of the random factor resulted in more realistic cell pattern in comparison to the pure mode. The cell divisions were analyzed statistically on the top view. When all of the division walls were considered, their angular distribution was uniform, whereas in the distribution that was limited to apical initials only, some preferences related to their arrangement at the dome summit were observed.

**Conclusions:**

The realistic surface cell pattern was obtained. The present method is a useful tool to generate surface cell layer, study activity of initial cells and their derivatives, and how cell expansion and division are coordinated during growth. We expect its further application to clarify the question of a number and permanence or impermanence of initial cells, and possible relationship between their shape and oriented divisions, both on the ground of the growth tensor approach.

**Electronic supplementary material:**

The online version of this article (10.1186/s13007-017-0262-7) contains supplementary material, which is available to authorized users.

## Background

The shoot apex is a meristematic organ that is located at the end of a stem. It consists of the apical part, usually shaped like a dome, which is called the shoot apical dome [1] and an underlying segment that includes the primordia of the leaves and flowers. The apical dome comprises the shoot apical meristem (SAM), which is the region that is dedicated to growth [2]. Through the lifespan of a plant all of the above-ground tissues of the plant are generated from this region. The growth of the SAM results from cell enlargement and division and these processes are coordinated so that shape of the apical dome does not change despite the constant flux of the cells from the meristem into the newly initiated lateral organs and inner parts of the stem [3].

The SAM is a self-organizing system [4] that performs two basic functions [2]—providing new cells that subsequently elongate and differentiate into the primary tissues of the stem and lateral organs and maintaining its shape. It harbors a small population of undifferentiating stem cells in its distal region that constantly renew themselves, supplying new cells for growth and tissue formation. The stem cell population includes the initial cells of the SAM, which are located at the pole of the shoot apical dome [2, 5]. In this paper we are interested in shoot apices with the tunica/corpus organization [1], which is typical for angiosperms, where the initial cells occupy two or more cell layers. Zea, for example, has a single layer of the tunica (L_1_) that overlaps the L_2_ layer of the corpus, while Arabidopsis has a two-layered tunica (L_1_ and L_2_) that overlaps the corpus. Each tunica layer and corpus has own initial cells [2]. These layers are clonally distinct and can be recognized genetically [6, 7]. We focus our attention on the layer in which the initial cells and their derivatives divide perpendicularly to the surface, thereby giving rise to the epidermis.

The initial cells do not differ from their nearest derivatives morphologically and they are identified mainly by their position at the pole of the apical dome [2]. The number of initial cells in the surface layer is not clear. A triad of initials that meets at the dome tip is regarded to be stable geometrically [8, 9], but clonal analysis has shown [10] that their number ranges from 1 to 4. Moreover, the initials can generally be impermanent [10, 11], but even in the simplest case of three permanent initials we do not know how they grow and divide to produce the entire surface cell layer through further divisions of their derivatives. Because direct empirical investigations are extremely difficult, computer modeling seems to be the best way to fill this gap.

The meristematic tissue can be visualized as a three-dimensional meshwork in which the filaments of the mesh are the contact edges of the cells. During growth, the meshwork is extended and deformed and new cells that result from cell divisions are generated within the spaces previously occupied by the parent cells. After many generations of cell divisions, the cellular clones can be recognized. The clones that are observed at the dome surface are arranged into cell packets which are distinguished by thicker walls [12, 13]. Analysis of the shape and dimensions of these cell packets provides valuable information about the surface growth, i.e. the directional variation of the relative elemental rate of the linear growth (R_l_) in the plane tangent to the surface. Observations have shown [10, 14] that the surface growth in the central region is nearly isotropic (locally the values of R_l_ in all of the directions calculated in the plane tangent to the surface are the same) and becomes anisotropic at the lateral surface of the dome. In vivo investigations using the replica method [15, 16] and confocal laser scanning microscopy [3, 17–19] support this view.

The shoot apex, like other plant organs, grows symplastically [20, 21]. Symplastic growth is the coordinated growth of cells during which the neighboring cells do not slide or glide with respect to each other, thereby preserving their mutual contacts. Such growth is of a tensor nature [22–24]. In theoretical studies, it can be conveniently generated by a second rank operator, which is called a growth tensor, GT [23]. Examples of GT-based growth were described in application to differently shaped shoot apices [14, 25–27], including the case in which the surface growth was isotropic.

Many computer models have been formulated to simulate the growth of plant tissue (reviewed by Prusinkiewicz and Runions [27]). When growth is accompanied with cell divisions, they implement particular rules about how to orient the division walls. On the one hand, the orientation of the cell division depends on the geometry and extension of the cell [28–30]. On the other hand, the primary role of the directional signals that result from the growth and mechanical stress has been postulated [31–35]. The Errera’s hypothesis, which postulates that cells divide along the smallest division plane, is regarded as the most universal for plant tissue [36]. The examples of its application to generate the development of the surface cell pattern of the shoot apex were described [34, 35, 37, 38]. However, little is known how this rule works in the application to the dome-shaped apex considered in the 3D, and the cells which are postulated to be initials from which the entire surface cell layer of is originated.

This paper shows how to generate the surface cell layer in 3D, using the simulation model for growth based on the growth tensor. The paraboloidal-shaped shoot apical dome in which the cell layer originates from three initial cells that are located at the dome summit is considered. These initials, described by polyhedrons develop through growth and cell divisions under the isotropic surface growth. The cells divide anticlinally with the smallest division plane. Two modes of a location of the plane are tested—without and including a random factor that slightly affects the position the plane with respect to the geometrical cell center. The formation of the virtual surface cell layer is described. We focus our attention on the surface cell pattern, the initial cells, and the fates of cellular clones that are derived from them. The orientation of cell divisions is analyzed statistically. The method and its potential applications are discussed.

## Methods

### Shoot apical dome and initial cells

**Fig. 1:**
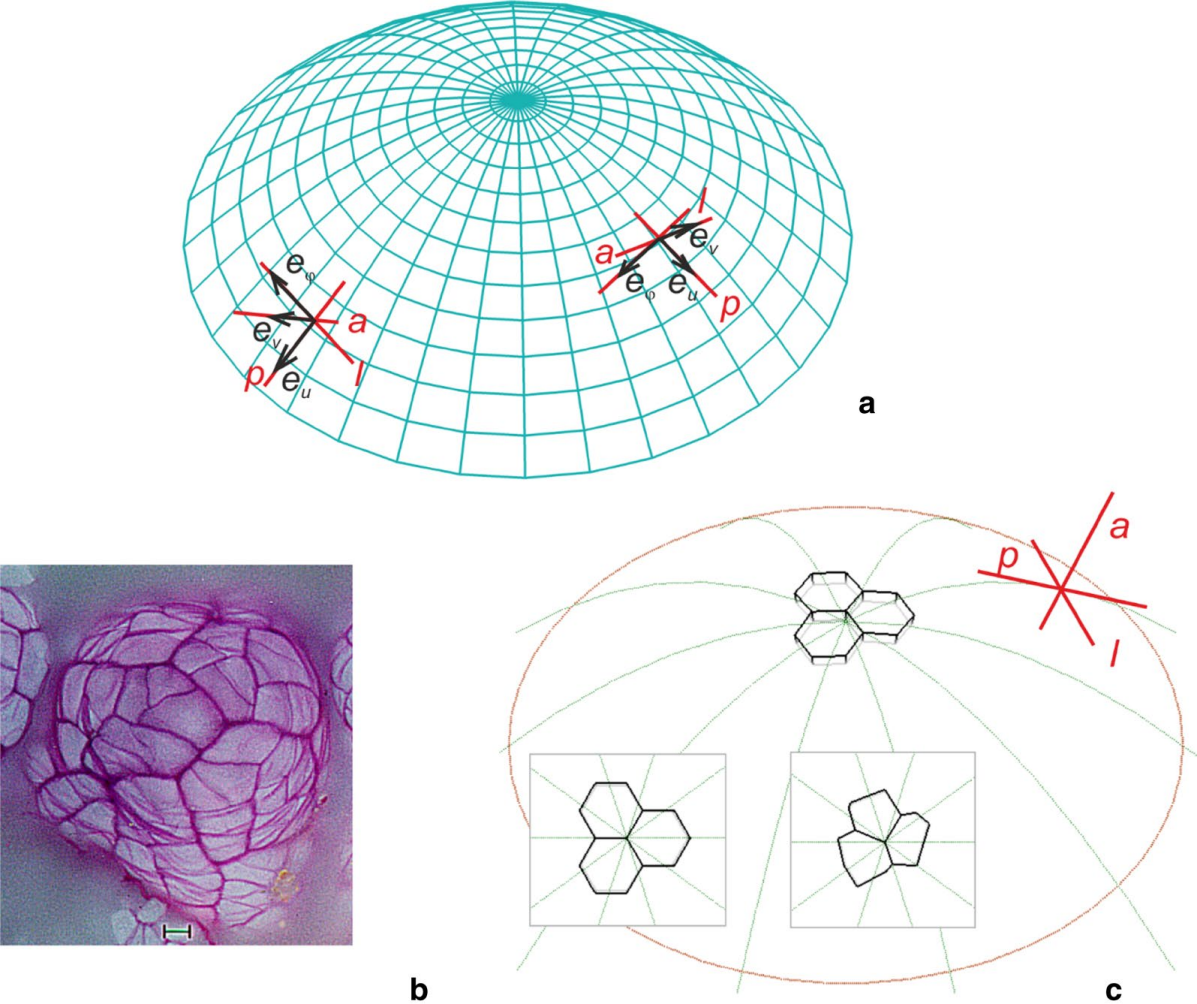
The shoot apical dome modeled in this paper and the cells assumed to be initials of the surface cell layer: **a** the dome surface visualized by the coordinate lines of the paraboloidal system (*u*, *v*, *φ*); at the exemplary point unit vectors **e**_*u*_, **e**_*v*_, **e**_*φ*_, respectively, represent three mutually orthogonal directions: *p*-periclinal, *a*-anticlinal, *l*-latitudinal; **b** *Picea abies* microphotograph showing a triad-type cellular pattern with clear apical initials observed in a seedling at the age of about 12 plastochrons [65], **c** position of the exemplary initials at the dome summit and two triads of the initials composed of the uniform and not uniform cells (insert shows top view) assumed in the modeling. At the dome surface meridional growth trajectories (green), the *p*, *a*, *l* directions (red) and the boundary of the simulation area at the dome base (brown) are indicated

The modeled apical dome has a paraboloidal shape and grows steadily without a rotation around the symmetry axis. In the paraboloidal coordinate system (*u*, *v*,*φ*), the dome surface is represented by *v*_*s*_ = 5.0 (Fig. 1a) and three unit vectors—**e**_*u*_, **e**_*v*_, **e**_*φ*_, respectively, represent the periclinal (***p***), anticlinal (***a***) and latitudinal (***l***) directions. Let us assume that the three cells that meet along the common edge that passes through the dome axis (Fig. 1b, c) are the apical initials of the surface cell layer. These initials, which are described by polyhedrons, are arranged so that their common edge passes through the dome summit (the central point of the paraboloidal curvature). They are given by the vertices situated on the surfaces *v*_*s*_ = 5.0 (inner) and *v* = 5.2 (outer). Two triads of apical initials will be used as the incoming data in the simulation. One (In1) includes uniform cells that have the same shape and size, whereas the other (In2) consists of cells that differ in shape and size. The entire surface layer will be generated from these initials.

The formation of the surface cell layer is visualized on both the side and top views (Fig. 1b, c). The top view is a projection of the layer on to the plane tangent to the surface at the dome summit. In this view, all of the displacement lines that are parabolic-shaped are seen as radii (inserts in Fig. 1b, c), whereas the directions ***p***, ***a***, ***l***, which are mutually orthogonal, are represented by the cross.

### Growth field

**Fig. 2:**
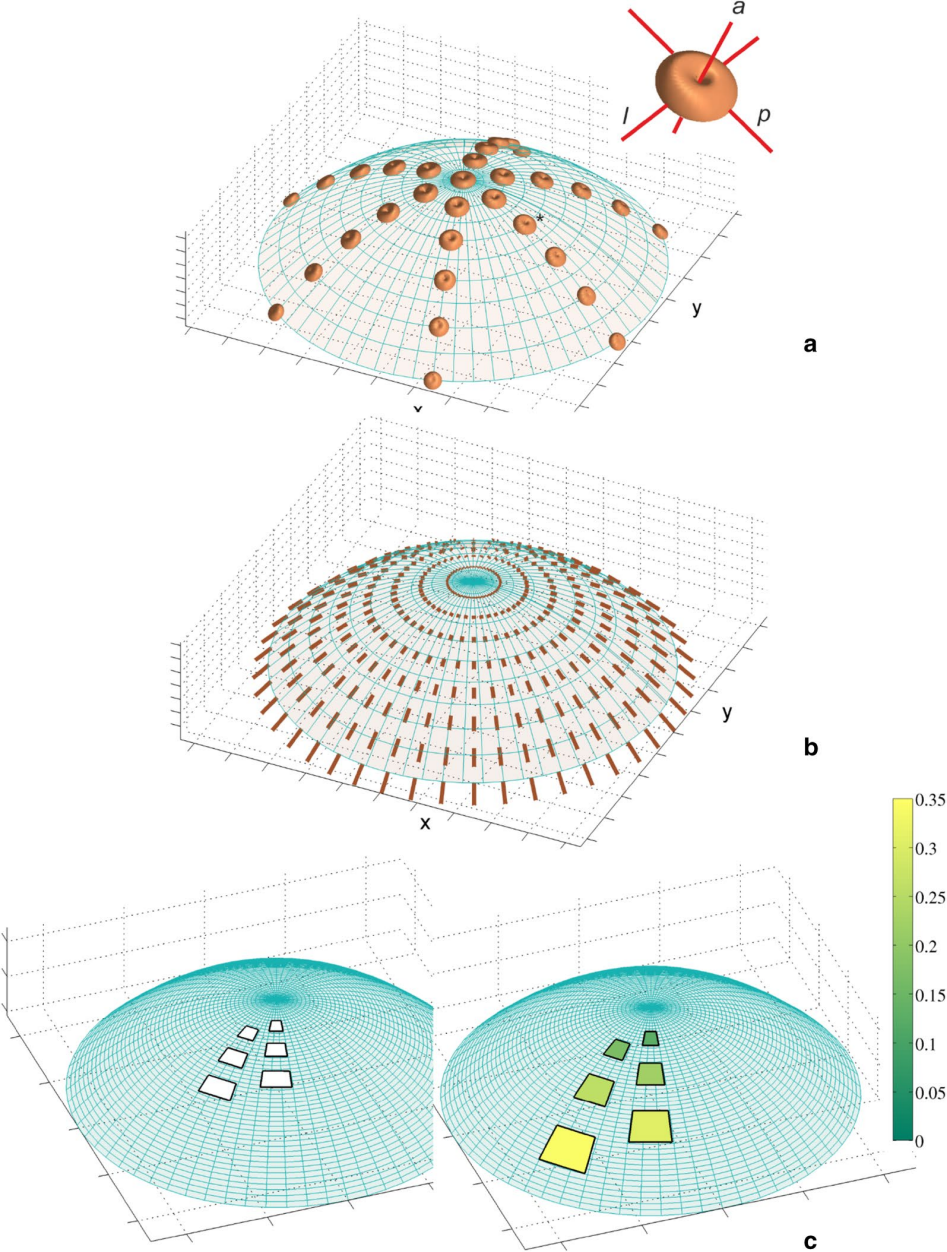
The growth field assumed for the apical dome surface: **a** distribution of the relative elemental rate of the linear growth (R_l_) visualized by the 3D indicatrices; at each point, the value of R_l_ in a given direction is proportional to the distance from the point to the indicatrix surface along this direction; the insert shows the orientation of the exemplary indicatrix (asterisk) with respect to the directions *p*, *a*, *l*—notice the isotropic surface growth (values of R_l_ in the plane tangent to the surface are the same). **b** The displacement velocity field, the **V** vectors are represented line segments; **c** the deformation of exemplary rectangles during growth, the relative rate of growth in area is color-coded. The surface growth decreased basipetally simultaneously causing an increase in both the **V** and the relative rate of growth in the area

The GT field that defines the isotropic surface growth of the apical dome was adopted from previous studies [14, 26, 39]. For the GT matrix and the equations used to calculate the growth rates, see Additional file 1. The spatial and directional variation of R_l_ at selected points of the dome surface is demonstrated by the 3D plots in Fig. 2a, which are called indicatrices [12, 40]. At each point, the R_l_ in a given direction is proportional to the distance from the point to the indicatrix surface along this direction. Notice how the values of the rate change through the dome surface. Those along ***p***, ***l*** and the other directions that lie in the plane tangent to the surface reach a maximum in the very apical region and decrease successively with their distance from the summit. The R_l_ along ***a***, which is much smaller in comparison to the previous ones, increases basipetally from the summit to the lateral surface. Because locally the values of the R_l_ calculated in all directions are the same in the plane tangent to the surface, the surface growth is isotropic.

### Cell expansion and division

In order to simulate cell expansion, the displacement velocities are necessary. In the paraboloidal system, the **V** vector has three physical components—V_*u*_, V_*v*_ and V_*φ*_. Both V_*v*_ and V_*φ*_ are equal to zero as a consequence of the assumption that the considered apical dome grows steadily and does not rotate around the symmetry axis. The third one was specified by the condition that ensured the isotropy of the surface growth (Additional file 1). After Hejnowicz et al. [26], we obtained $V_{u} = \frac{{c\left( {u^{2} + v^{2} } \right)}}{{\sqrt {u^{2} + v_{s}^{2} } }} u$, where *c* = *const*. How the assumed distribution of R_l_ relates to the displacement velocity field as well as the relative rate of growth in the area calculated for the exemplary rectangles that expand during growth is shown in Fig. 2b, c, respectively. Notice that all of the velocity vectors are tangent to **e**_*u*_ and that their lengths increase with their distance from the summit. Similarly, the area of the exemplary rectangles that was considered in the same time period increased basipetally. The relative rate of growth in this area (color coded) increased almost seven times compared to the fates of the two rectangles that were originally located at different distances from the dome summit.

The assumed velocity field caused that the cells were displaced only basipetally along the meridional growth trajectories that were appropriate for their positions. Knowing the coordinates of the cell vertices at *t*_0_, the new positions of these vertices at (*t*_0_ + *Δt*) were calculated from the old ones by integrating V_*u*_ with respect to time.

During growth the cells increased in volume and divided anticlinally according to the following rules:

1. A division occurred when the cell volume that was assumed to be critical was exceeded. Then, the parent cell was replaced by two daughter cells, both of which were represented by polyhedrons.
2. The cell division was defined by a criterion of the smallest division plane (SAD). This plane was implemented assuming one of the two locations of the plane within the cell. In mode I, the plane passed through the geometrical cell center (C). In mode II, a spherical region with a small radius *ρ* around the center was established and the plane passed through point M, which was defined randomly within this region. In both, the plane resulted from the calculation of 360 potential division planes that either passed through points C (mode I) or M (mode II) every one degree. The mode, which was specified at the beginning, was used for all of the cells in a given simulation.
3. After formation, the division wall was slightly shortened by *k* percent of its former length due to the difference in strength between the walls of mother cell and the division plate. The difference is suggested to yield a perpendicular junction, whose new plate gains strength, thus allowing the three facets to rearrange to form angles that tend to be equal [41]. This shortening resulted in a redefinition of the angles between anticlinal walls at both of the newly formed anticlinal edges.

**Fig. 3:**
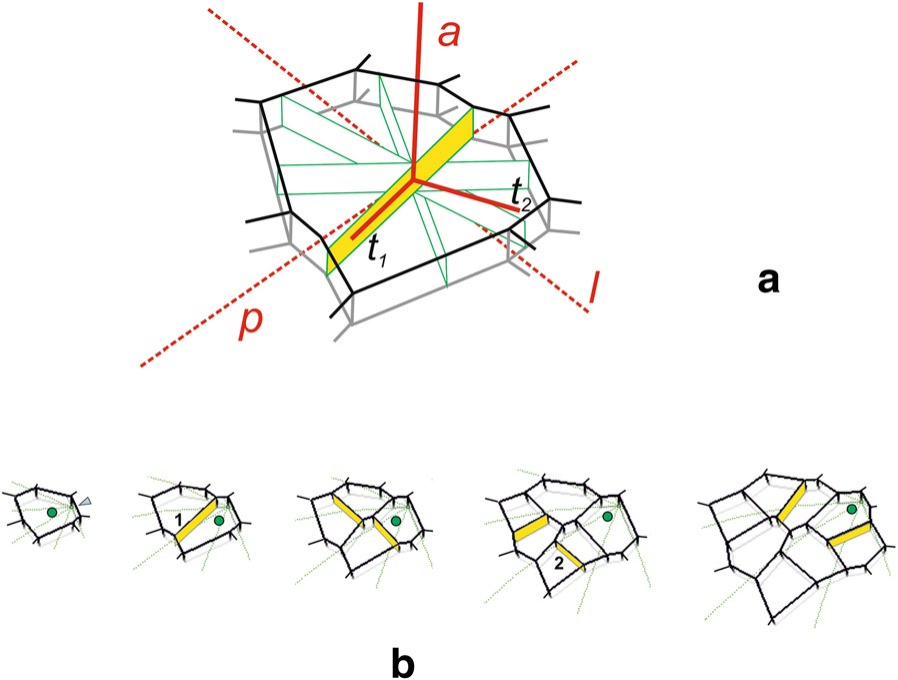
The SAD algorithm for the shortest anticlinal division (**a**) and temporal sequence showing its application (**b**) to generate growth and exemplary divisions of the apical initial and its nearest derivatives. In **a**, among many possibilities of anticlinal divisions (green), the wall with the smallest division plane (yellow) was selected. This plane, defined by ***a*** and ***t***_**1**_ is perpendiculat to ***t***_**2**_. In **b** the initial (circle) renews itself and maintains contact with the dome summit (arrowhead), whereas all of the derivatives are displaced basipetally further away from the summit during growth. All of the cells divide perpendicularly to the surface along the shortest division plane but with different the orientation with respect to meridional growth trajectories (green). The walls 1 and 2 result from more or less the meridional and latitudinal divisions, respectively, whereas the remaining walls result from the oblique ones

The way in which the above rules work can be seen in Fig. 3b where the exemplary divisions of the apical initial and its derivatives are presented. At the beginning of the simulation, the initial that was assumed at the input increased and maintained contact with the dome summit and soon divided into two daughter cells, reaching the volume that was necessary for division. The two daughter cells were created by the division of the wall with a meridional orientation. The one that had contact with the summit became the new apical initial, while its sister was displaced away from the summit during further growth. Next, both the cells expanded, divided by more or less similar transversal divisions and the cell tetrad was obtained. Like before, the cell that maintained contact with the summit became the new apical initial, while the three remaining ones formed a group of cells that changed their position in successive steps of the simulation. During further development all of the cells increased and the following divisions took place, at first in the cells that occupied the proximal region, and then those in the distal region. Finally, there were eight cells in the cell pattern—one apical initial and seven derivatives of the initials that had previously functioned. A total of three divisions of the initial cell were observed, which means that the apical initial that was assumed at the input renewed itself three times. In this sense, all of the derivatives were derived from the same initial lineage.

Only anticlinal divisions were generated, but with different orientations. Taking the meridional trajectories as the reference, walls 1 and 2 resulted from anticlinal-meridional and anticlinal-latitudinal divisions, respectively, while all of the remaining walls resulted from the anticlinal-oblique ones. Because in our model there were no other divisions than anticlinal ones, the term ‘anticlinal’ will be omitted in order to simplify the description. Notice that all of the newly formed walls were slightly shortened as the result of application of the rule 3, but there was never a change of their orientation.

### Simulations

Simulated growth was obtained through the operational application of the GT field to the meshwork that represented the three apical initials that were assumed at the input. The application was such that the symmetry axis and the v_0_ surface of the GT field coincided with the symmetry axis and the v_0_ surface of the apical dome and this coincidental situation was preserved over time. Using these assumptions, steady-state growth was generated, which means that the fates of individual cells were only determined by the positions of the cells in the GT field.

The temporal sequences of the formation of the surface cell layer were obtained using the iteration method (see Additional file 2: Figure S1). Each simulation included 3500 time-steps with Δt = 0.001. The parameters of the model were as follows. Velocity V_*u*_ was specified by c = 1. The volume of the initials In1 at t_0_ was assumed to be critical for cell division (vol_cr_). In mode I the division plane passed precisely through the geometrical cell center, whereas in mode II it passed through point M, which was found randomly within the spherical region surrounding this center that had a radius equal to 2% of the distance from point C to each anticlinal wall of the initials In1. After division, the newly formed wall automatically decreased by 7% of its former length.

The cell wall meshwork at the beginning of the simulation, which included only the apical initials, became larger and larger in successive steps. It was necessary to establish a boundary for the simulation area at the dome base. The boundary was defined by the intersection of the dome surfaces *v*_*s*_ = 5.0 and the surface *u* = 5.2, which was oriented anticlinally. The cells that were displaced beyond the boundary were omitted from the print-out. After this boundary was reached, the meshwork stabilized at the level of N = 337 ± 15 cells. From that point on, the number of omitted cells was more or less at the same level as the number of newly formed cells resulting from the divisions that were taking place within the whole simulation area.

### Analysis of division walls

In the course of the simulation, about 1700 cell divisions occurred for In1 and 1550 cell divisions occurred for In2 (each simulation included the same number of time-steps, but initials In1 began to divide at once, whereas In2 began to divide successively from the 79th to 153th time-steps of the simulation). All of the division walls were analyzed for their spatial distribution and angular variation from the top view. Moreover, using the cell pattern obtained at the end of the simulation (top view), the divisions that were formed during the final 20 time-steps were also marked. For the angular variation, immediately after the creation of a new wall, its angle was measured with respect the radius and assuming 0° for the radial direction. The angles had different values within the range (− 90°, + 90°). The walls from the range (− 15°, 15°) were regarded as more or less meridional, while those from two ranges (− 90°, − 75°) and (+ 75°, + 90°) were regarded as latitudinal and the remaining ones as oblique. In addition, using an original program that was written in MATLAB (Matworks), the division walls were collected together, divided into classes every 10° and arranged into a frequency diagram. Next, the angular distributions of the division walls that were obtained in different simulations were described statistically. To test whether there were differences in the angular variation between particular simulations, two non-parametric tests, the Kruskal–Wallis H test and U Mann–Whitney test, were used. The Gaussian mixture model was adjusted for the angular distribution of the division walls. The hypothesis that the cells divided into two equal daughters, was verified using the *t* test. All of the statistical analyses were performed with STATISTICA version 12.0 (StatSoft Inc. 2014).

## Results

**Fig. 4:**
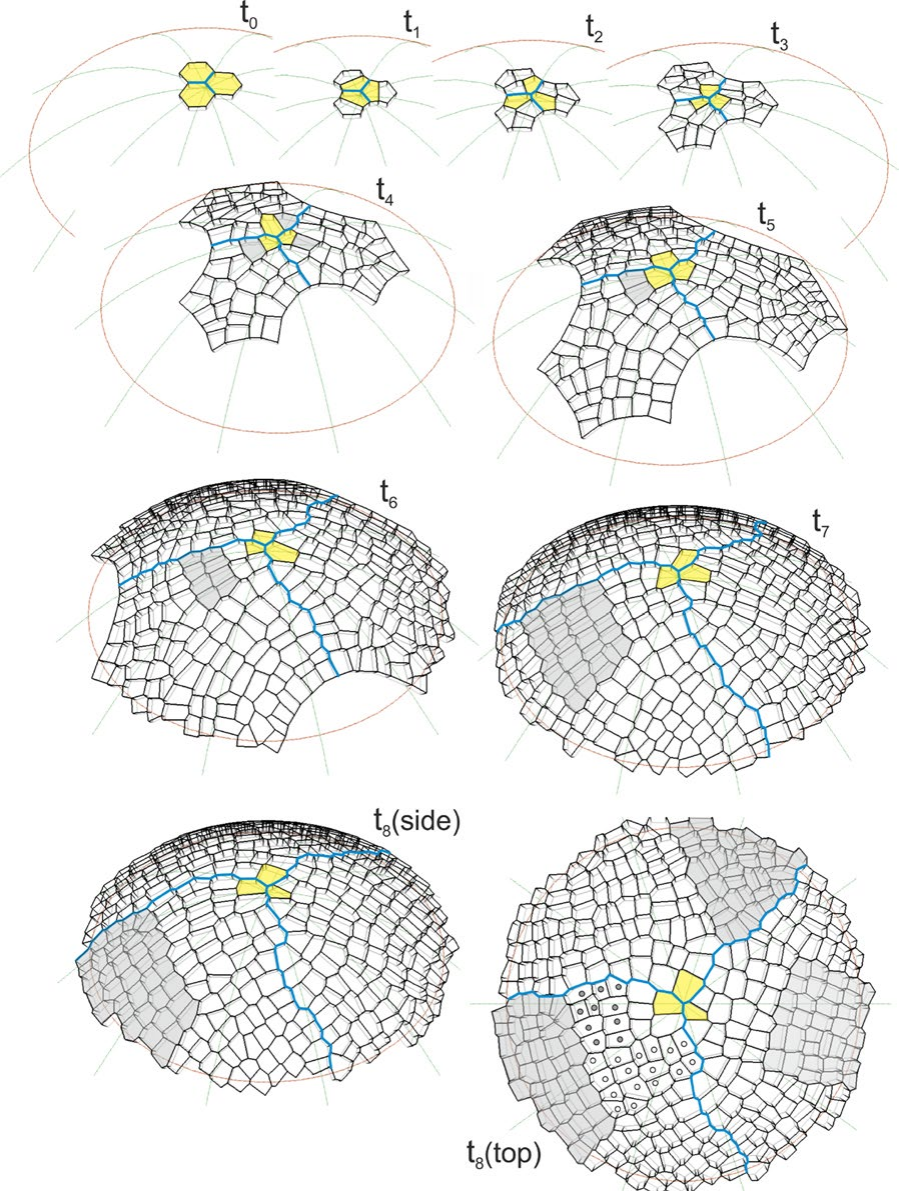
The computer-generated sequence of the formation of the surface cell layer of the apical dome. In the model the uniform initials (In1) and divisions with the SAD rule in mode I were assumed. The times from *t*_0_ to *t*_8_ (side view) show the development of the cell pattern; the apical initials (yellow), zigzag boundaries between sectors (blue) and one of cellular clones (gray) formed in the frontal sector are marked. At *t*_8_ (top view) the other clones considered in the text are indicated with respect to the previous one, those in the frontal sector (open and closed circles) are younger and those in the remaining sectors (gray) are similar in age

The sequence of the formation of the surface cell layer of the apical dome is shown in Fig. 4 and the animation (Additional file 3: Video S1). In the simulation, the initials In1 and cell divisions with the SAD rule in mode I, i.e. those with the smallest division plane passing through the cell center, were assumed. Let us consider how the cell pattern of this layer originated, formed and developed in the successive stages.

The initials In1 were uniform in their shape and size, and therefore, they grew and divided in the same way. At t_1_, after latitudinal division, three pairs of similarly shaped daughter cells were obtained. The three cells that maintained contact with the dome summit along a common edge became the new apical initials and each of their sisters became the clonal derivatives. At t_2_, all of the cells divided via meridional divisions and three similarly arranged cell tetrads were obtained. Like before, the cells that met at the dome summit became the new initials, while the remaining cells enlarged the population of derivatives. In the following stages, the cell meshwork increased successively reaching at t_3_-24, at t_4_-75 and at t_5_-156 cells. At the same time, some differences between the derivative sectors appeared in the cell patterns. They resulted from the slightly different orientations of the division walls and the increasing number of cells in the population of derivatives. At t_6_ the number of cells in the meshwork increased so much that the majority of the cells deriving from the sisters of three initials that were established at t_1_ exceeded the simulation border. Starting from t_7_, the surface cell layer was complete. During further development, its cell number stabilized at the level of almost 350 and the cell pattern could be considered to be self-perpetuating.

Three derivative sectors that were separated by zigzag boundaries (blue) could be recognized in the cell pattern of the surface cell layer. The boundaries changed dynamically over time as a result of the continuous flow of cells from the distal portion of the apex, although the sectors were more or less similar in the occupied part of the dome surface. At any given time, each sector included the apical initial, its sister and a number of cell packets that were derived directly from the sisters of the initials that had functioned previously. To show how these cell packets were formed, let us consider the frontal sector (Fig. 4), for example, at time t_3_, in which the frontal sector included the apical initial (yellow) and seven clonal derivatives. At t_4_, the initial renewed itself (yellow) and the cell packet that was observed in the following times originated from its sister (gray). The packet increased successively to 2 (t_5_), 8 (t_6_) and 30 (t_7_) cells, while simultaneously moving away from the summit. Finally (t_8_), it became so large that only its distal part remained in the simulation area. At t_8_ (top view) other cell packets were also observed. Three younger, and thus smaller than the gray one, occupied the distal region of the sector. Those marked by open and closed circles derived from the initials that were renewed in the period from t_4_ to t_6_ (see the animation Additional file 3: Video S1), the third one (three cells that were adjacent to the initial and its sister) was the youngest. The next three cell packets, which were older than the gray one, were in this sector. Two of them, which originated from the sisters of the initial that was defined at t_1_ and t_2_, left the simulation area, while only a small part that was situated in the lateral region of the sector remained from the third one, which was initiated at t_3_. Notice that all of the cell packets have an interesting alignment within the sector. Taking their origin into account, the divisions of the renewed apical initials are manifested in their shape and orientation.

Two large cell packets in the neighboring sectors (t_8_, top view) were the same age as the gray one in the frontal sector, and therefore, they developed at a similar distance from the dome summit. The packet in the sector on the right looks similar in its shape and cell wall system to the previous one in the frontal sector—its cell pattern was regular and the domination of cell walls with a meridional and latitudinal orientation could be observed. The packet in the sector at the top, in contrast, has differences in its shape and alignment within the sector and in its cell pattern there occur oblique cell walls which are relatively frequent. These differences may relate to their origin (t_4_ in Fig. 4 side view). The cell packets in the sectors on the left and right resulted from latitudinal divisions of their apical initials, whereas the one on the top resulted from meridional divisions of its apical initial.

**Fig. 5:**
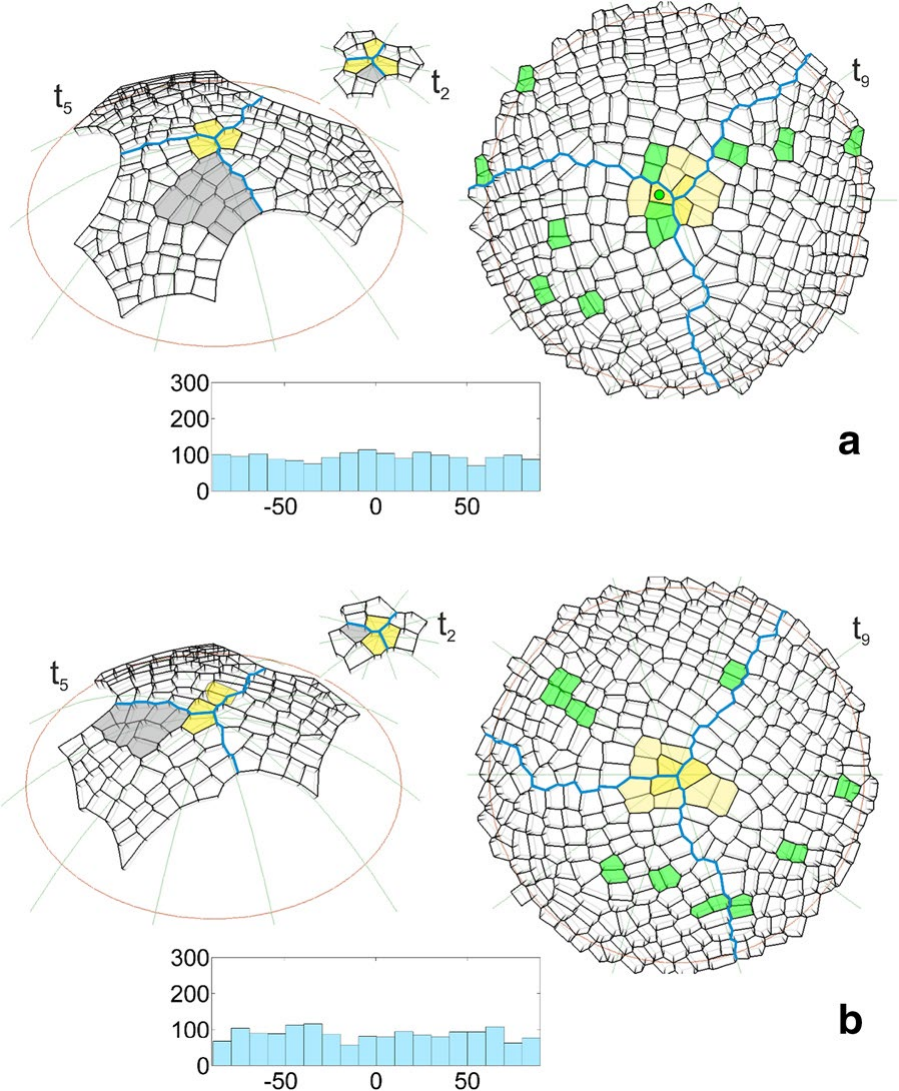
Selected times showing the results of two simulations in which the surface cell layer originated from **a** uniform or **b** not uniform apical initials, assuming cell divisions with the SAD rule in mode I in both. Times t_2_ and t_5_ (side view) like in Fig. 4, t_9_ (top view) shows a further developmental stage that was not considered earlier. In the cell pattern there are marked: the apical initials (yellow), the boundaries between the sectors (blue) and the cellular clone initiated at t_2_ by the initial sister (gray). At t_9_, the cell divisions are indicated in the apical initials (the light yellow cell packet formed by the two final self-renewing cells) and other cells (green) during the following 20 time-steps at the end of the simulation. The diagrams shows the angular distribution of all of the division walls (top view, where 0° and 90°, respectively, represent meridional and latitudinal orientations)

The uniform initials In1 were used in the simulation described above. It was interesting to repeat the simulation assuming the initials In2, which were not uniform in shape and size. The formation of the surface cell layer that originated from these initials can be seen in the animation shown as Additional file 4: Video S2. In Fig. 5 some of the results that were obtained for both initials In1 and In2 are compared on the examples of the times t_2_ and t_5_ like in Fig. 4 and t_9_ which shows a further developmental stage that was not considered before. We focused our attention on the cell pattern as well as the distribution and angular variation of the division walls. At the beginning of the simulation, the initials In1 grew uniformly and divided in a similar way (Fig. 5a). In the case of the initials In2, this was not so, because after one meridional and two oblique divisions, all of the cells divided by a latitudinal division and the cell tetrad was not as uniform as the one obtained previously (Fig. 5b). Such divisions caused the differences in the shape and dimension of the corresponding sectors to be significant at t_5_. For initials In1, all three sectors had a similar shape and included the same number of cells (Fig. 5a). For initials In2, they were smaller, differently shaped and included a smaller number of cells (Fig. 5b). Other differences were related to the cell pattern. Notice the gray cell packet. For the uniform initials, it was situated on the right side of the frontal sector (Fig. 5a), whereas for the initials that were not uniform, it was on opposite side of this sector (Fig. 5b), which caused another cell to become the apical initial at t_2_. At t_9_ (top view) where the entire surface layer is demonstrated, both cell patterns look similar, and the slight differences relate to the clonal boundaries between the sectors and the cell wall arrangement. Let us consider the small cell packets (yellow) surrounding the dome summit, in which the last two divisions of the renewed initials were manifested. All of the packets for initial In1 resulted from a combination of longitudinal (meridional) and transversal (latitudinal) divisions (Fig. 5a). For initial In2 such a combination occurred in two cases, the third cell packet resulted from transversal division first and then oblique divisions (Fig. 5b). In the cell pattern that was considered, the cell divisions (green) that were observed at the end of the simulation (during the final 20 time-steps) are indicated. For both types of apical initials, they were distributed randomly through the surface with similar amounts and proportions between the sectors (3:3:5 in Fig. 5a and 3:3:3 in Fig. 5b). The histograms of the angular variation of all of the division walls (generated during the whole simulation) indicated uniform distributions for both In1 (Fig. 5a) and In2 (Fig. 5b). The U Mann–Whitney test gave Z = −1.4, p = 0.16, thus showing the absence of a statistically significant difference between them.

**Fig. 6:**
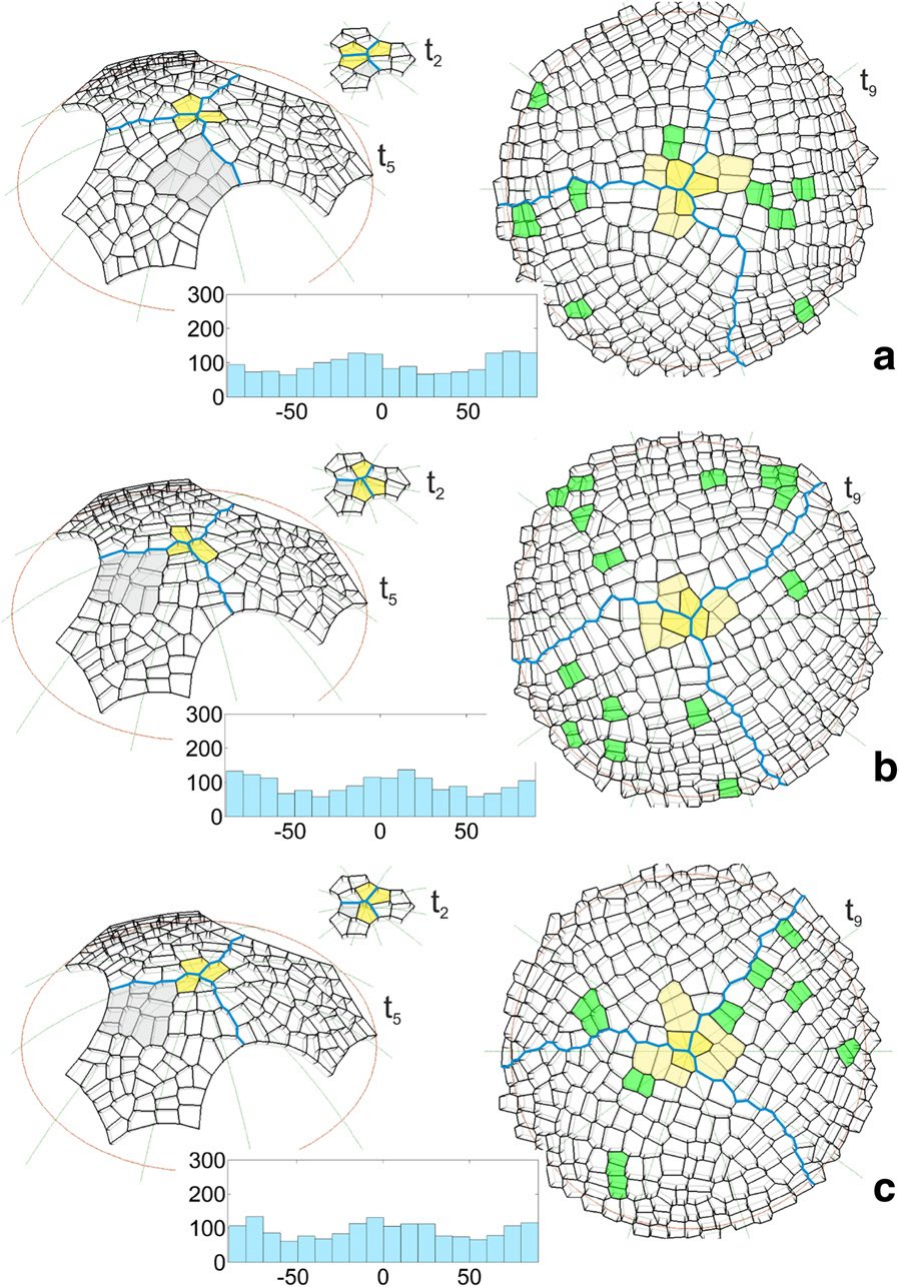
Cell patterns and diagrams of the division walls obtained for the uniform apical initials (In1) like in Fig. 5a, but in the three simulations (**a**–**c**) in which the SAD rule was applied in mode II, i.e. the cells divided along the smallest division plane that did not pass through the cell center. For another explanation see Fig. 5

**Fig. 7:**
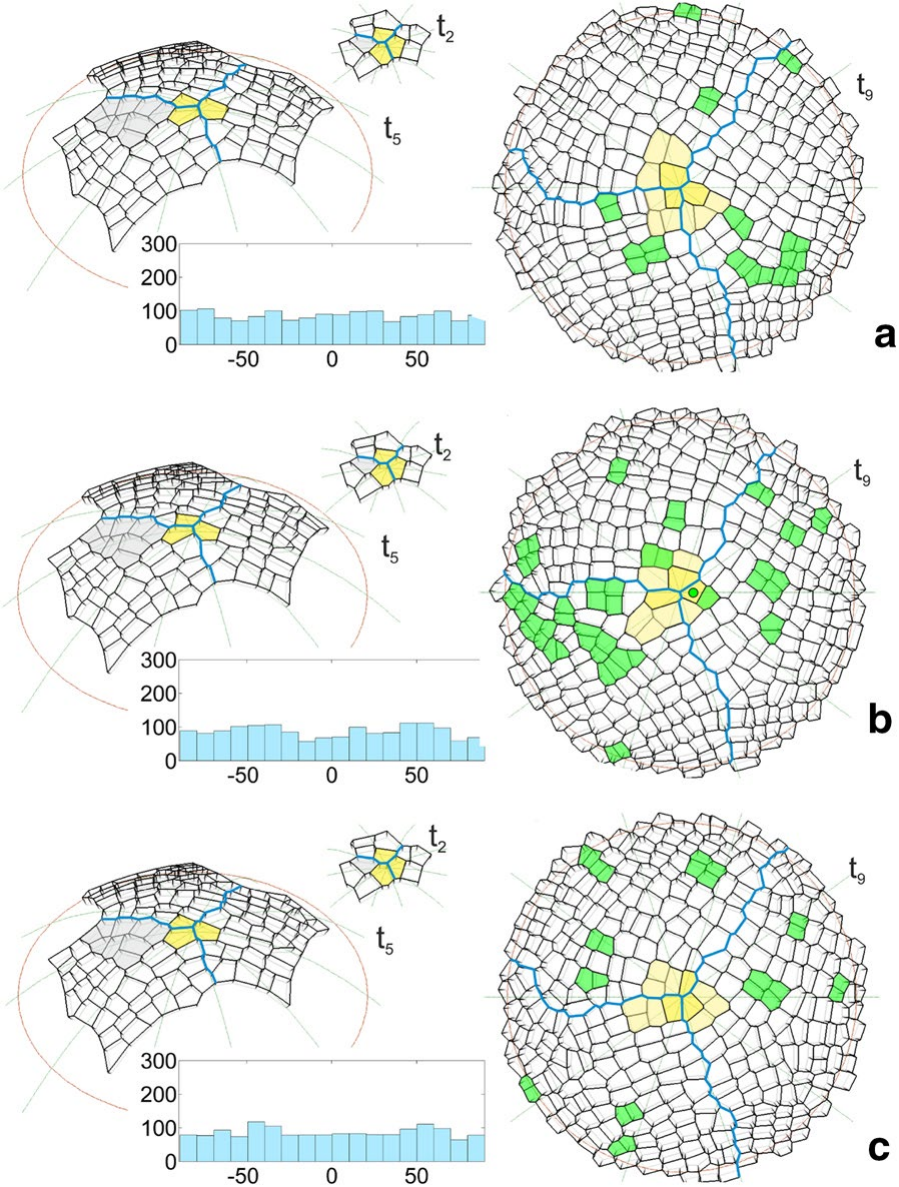
Cell patterns and diagrams of the division walls obtained for the not uniform apical initials (In2) like in Fig. 5b, but in the three simulations (**a**–**c**) in which the SAD rule was applied in mode II, i.e. the cells divided along the smallest division plane but did not pass through the cell center. For another explanation see Fig. 5

The results described above were obtained assuming the SAD rule in mode I. Figures 6 and 7 show the application of this rule in mode II in which the location of the division wall was affected by a random factor with respect to the cell center. This means that like before, walls that had a minimal division plate were generated, but did not have to pass through the cell centers. The application of such a factor caused every repetition of the simulation with exactly the same parameters to result in a slightly different cell pattern at the corresponding times of the surface layer formation.

Figure 6a–c and the animation Additional file 5: Video 3 show the results of three simulations in which the initials In1 were assumed. As was expected, in all of the simulations the clonal sectors at corresponding times developed like the one in Fig. 5a, in which the cell divisions in mode I were assumed. Some differences did appear in the details of the cell patterns. At t_2_ different configurations of three apical initials were already observed. They affected the further development and, in particular, led to changes in the location of the gray cell packet in the frontal sector. At t_5_, it was located on the right side of this sector only in Fig. 6a, but like before in Fig. 5a, in both of the remaining cases the cell packet occupied the left side (Fig. 6b, c). Moreover, the two cell packets that occupied the same position differed from each other in the details of cell patterns; the cell packet in Fig. 6b appears to be a mirror reflection of the one in Fig. 6a. At t_9_ (top view), the clonal sectors were more or less similar in relation to the occupied part of the surface, but when particular simulations are compared, there are differences in the details of the zigzag boundaries between sectors. Moreover, the small cell packets (yellow) surrounding the dome summit that resulted from the final two divisions of the renewed initials differed from one another. Only in Fig. 6a did these cell packet results from mutually orthogonal divisions, like earlier in Fig. 5a. In the two remaining cases, parallel (Fig. 6c) and oblique (Fig. 6b) division walls can be observed. Notice the distribution of the cell divisions (green) during final 20 time-steps of the simulation. Like in Fig. 5a, b, they are distributed randomly through the surface layer, but with more pronounced differences between the number of divisions in particular clonal sectors in one of the three simulations (2:4:4 in Fig. 6a, 8:1:6 in Fig. 6b and 2:5:3 in Fig. 6c). The three diagrams of the angular variations of the division walls (Fig. 6a–c) are not as uniform as earlier in Fig. 5a. In each simulation, two small maxima with distances of 80°–100° occurred. Comparing the histograms, these maxima occupy slightly displaced angle ranges; however, their occurrence is evident. For the Kruskal–Wallis test, which was applied to the three samples that were considered, we obtained H (2, N = 5103) = 4.72, p = 0.09, which means that there are no statistically significant differences between them. However, by comparing each of the three distributions with the one from Fig. 5 using the U Mann–Whitney test, we obtained: Z = 1.73, p = 0.08 for Fig. 6a, Z = −1.33, p = 0.18 for Fig. 6b and Z = −0.36, p = 0.72 for Fig. 6c, which indicates that the observed maxima are too small to be statistically significant.

Figure 7a–c and the animation Additional file 6: Video 4 show the results of three simulations in which the cells divide like before in mode II, but in the surface layer that originated from the initials In2. In all cases, the formation of the surface layer was similar to the one for mode I (Fig. 5b). The clonal sectors developed in a similar way at successive times, although some differences, which were much smaller than the previous ones (Fig. 6a–c), were related to the cell patterns but only at further developmental times. At t_2_ all of the apical initials occurred in the configuration that was previously observed (Fig. 5b). Similarly at t_5_, each of the three gray cell packets was shaped and developed in the same region on the left side of the frontal sector like in Fig. 5b. Small changes were only observed at t_9_ (top view), where the cell pattern of the whole surface was demonstrated. The zigzag boundaries between the sectors ran along slightly different paths and the cellular patterns that were compared between corresponding regions had in a slightly different arrangement. The yellow cell packets that manifested the final divisions of the apical initials differed from each other but, in general, they were similarly shaped like earlier for the initials In1 (Fig. 6a–c). In all cases, the cell packet resulted from two almost perpendicular divisions. The cell divisions (green) that were generated in the final period of the simulation (20 steps) were randomly distributed like before, but the number of divisions was greater and they were distributed unequally between the sectors (2:7:3 in Fig. 2a, 5:7:13 in Fig. 7b and 6:4:3 in Fig. 7c). The angular diagrams of the orientation of all of the division walls indicated a uniform distribution. Considering all three distributions, there were no differences between them because in the Kruskal–Wallis test, we obtained H (2, 4683) = 3.09, p = 0.21. Similarly, the application of the U Mann–Whitney test to compare each of the three histograms with the one from Fig. 5b led to the results (Z = 0.34, p = 0.73 for Fig. 7a, Z = −0.64, p = 0.52 for Fig. 7b and Z = 1.10, p = 0.27 for Fig. 7c), which indicate the absence of any significant differences between all of the pairs that were considered.

**Fig. 8:**
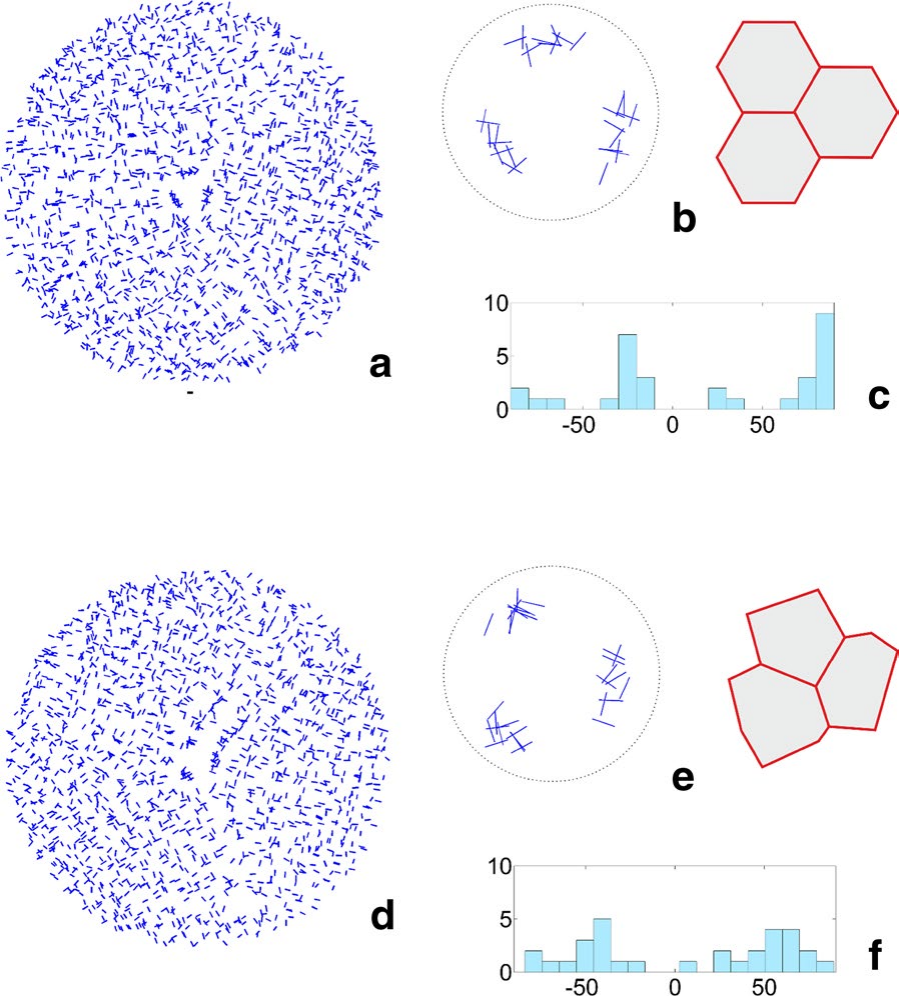
Distribution of the division walls of all of the cells (**a**, **d**) and apical initials only (**b**, **e**), in the case of the initials with the angular orientation of their walls (**c**, **f**). The results come from two simulations in which the surface cell layer originated from the uniform (**a**–**c**) and not uniform (**d**–**f**) initials (Fig. 5a, b, respectively). The distributions **a** and **d** show the picture as observed from the top view, but the walls are normalized to have the same length. In **b**, **e**, the normalized division walls are magnified to be the same scale as the incoming initials In1 and In2

In each angular diagram that was considered above, all of the division walls that were formed during the whole simulation were represented. It was interesting to extract the divisions of the apical initials from them and to study their orientations individually. In one simulation, three of the apical initials renewed themselves from 27 to 32 times, which means that there were 9–11 divisions per sector. The spatial distributions of the division walls of all of the cells and the apical initials only are shown in Fig. 8 on the example of the data from the two simulations presented in Fig. 5a, b (for the divisions of the initials in the remaining simulations—see Additional file 3: Video 1). We can see that all of the division walls were distributed uniformly through the dome surface in both the simulation that began from the uniform (Fig. 8a) and not uniform (Fig. 8d) initials. In contrast, the division walls of the apical initials occupied three specific areas that were situated at almost the same distance from the dome summit, more or less at the position of the initials that were assumed at the input (Fig. 8b, e). The angular diagrams for all of the division walls, which indicated a uniform distribution, were presented previously (Fig. 5a, b). The diagrams that are limited only to the apical initials are presented in Fig. 8c, f. For both types of data, two maxima occurred at − 30° and + 60° for the initials In1 (Fig. 8c) and at − 40° and + 60° for the initials In2 (Fig. 8f). Statistically, there was a significant difference between the data obtained for the initials In1 and In2; the results of the t test for independent samples was t = −2.57, p = 0.03. Similar maxima were also observed in the remaining simulations regardless of the SAD rule that was used (Additional file 3: Video 1). Obviously, the sample that only included the division walls of the apical initials (Fig. 5a, b) was much less numerous than the one for all of the cells (Fig. 8c, f). Nevertheless, we decided to compare them and obtained t = 2.33 with p = 0.02 for In1 and t = −0.12 with p = 0.91 for In2. Such results show that statistically significant differences only occurred in the case of the uniform initials.

Based on the histograms shown in Figs. 5a, b and 8a, b, it was possible to determine the percentage of oblique cell divisions with reference to the both meridional and latitudinal orientation taken together. For the divisions of all of the cells, there were 30% oblique division walls in the simulation with the initials In1 and 37% in the simulation with the initials In2. For the divisions of apical initials only, we obtained 15% oblique divisions in the simulation with the initials In1 and 52% in the simulation with the initials In2, which suggests that meridional and latitudinal divisions are more numerous when uniform initials are assumed as incoming data.

**Fig. 9:**
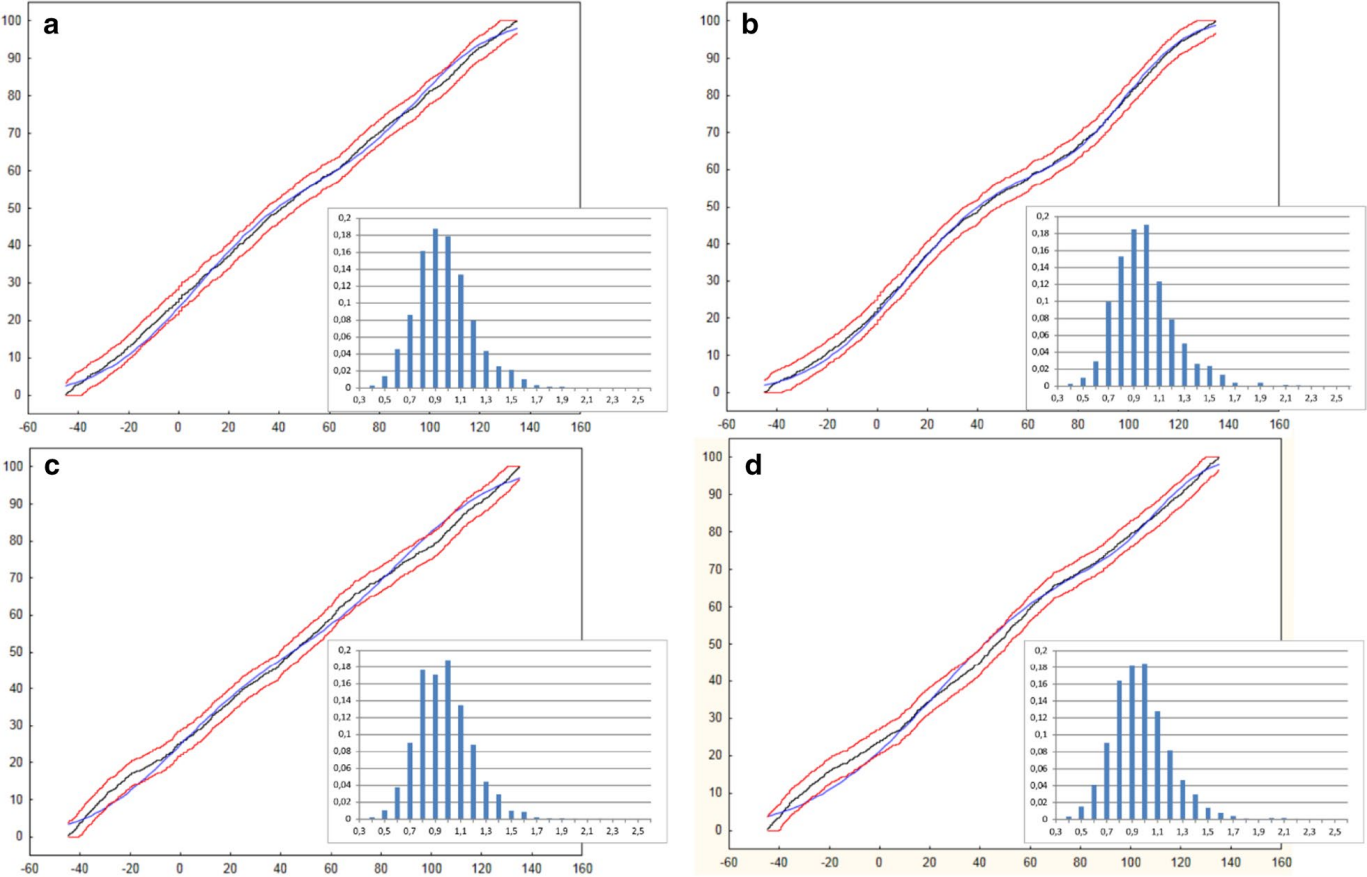
Statistics of the cell divisions in the exemplary simulations: **a** initials In1 and the SAD rule in mode I, data from Fig. 5a; **b** initials In1 and the SAD rule in mode II, data from Fig. 6b; **c** initials In2 and the SAD rule in mode I, data from Fig. 5b; **d** initials In2 and the SAD rule in mode II, data from Fig. 7a. Two distribution functions are shown—theoretical (black) and one describing the angular variations that were obtained in a specific simulation (blue), both at a 95% confidence interval (red). The histograms show the ratio of the volumes of the daughter cells, where 1.0 means exactly the same volume

In Fig. 9 some other results of the statistical analysis are shown. The angular variation of the division walls was described by a mixture of two Gauss distributions. A satisfactory fitting was only obtained for the uniform initials (In1). Using the Kolmogorov–Smirnov test we obtained d = 0.03, p = 0.06 for mode I and d = 0.01, p = 0.63 for mode II. Such results indicate a much better fit in the case of the simulation in which the orientation of the cell divisions was affected by a random factor. The histograms of the ratio of the daughter cell volumes are presented in the same figure. The volumes were consistent with the normal distribution and the sample mean was located in the range of 0.9–1.1, which indicates that the cell divided symmetrically to the first approximation. The lack of any differences between the volumes of the daughter cells was supported by the Mann–Whitney test. Comparing the cell divisions in mode I and II, we obtained Z = −1.51, p = 0.12 for the initials In1 and Z = −0.36, p = 0.71 for the initials In2. When both the uniform (In1) and not uniform (In2) initials were compared and assuming the cell divisions in the mode I, we obtained Z = −0.71, p = 0.86. Such results indicate a high degree of similarity in the volumes of the daughter cells.

**Fig. 10:**
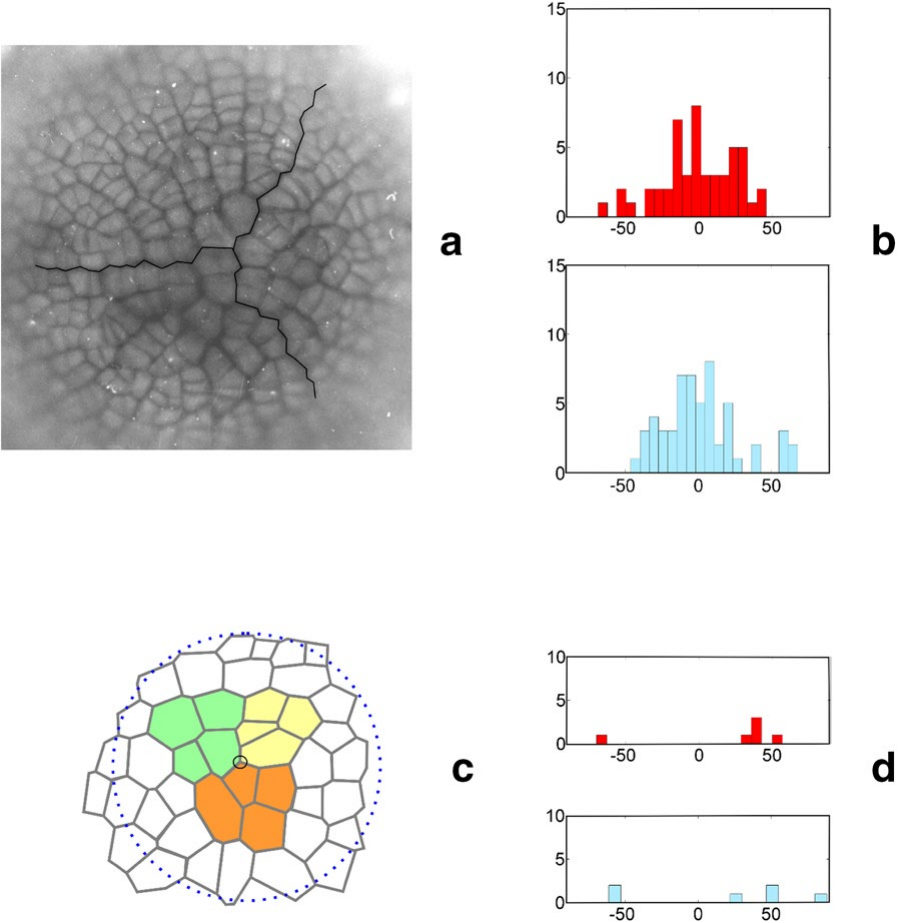
Comparing of the simulation results to empirical data **a** *Picea abies* microphotograph coming from surface cell pattern of the shoot apex of the tree several years of age (Nakielski—unpublished materials); the most likely boundaries between three clonal sectors are indicated, **b** diagrams of angular variation of the cell walls forming the zigzag boundaries between sectors in the real (top, data from **a**) and virtual (down, data from Fig. 6a) shoot apex, **c** the surface cell pattern in the central region of the *Arabidiopsis* shoot apex (redrawn from Willis et al. [42]); three cell tetrads that meet at the point which may correspond to the dome tip are marked, **d** diagrams as in **b** but showing angular variation of division walls formed by potential initials of the real apex (top, data from **c**) and initial cell of the virtual apex (down, data from t_9_ in Fig. 7a). For a given wall seen in the top view its angle with respect to the radius was measured

Some results related to the surface cell pattern obtained for the computer-generated apical dome were compared to those obtained for real shoot apices. In all computer-generated cell patterns the zigzags between clonal sectors manifested three-fold symmetry. The similar symmetry and generally radial organization of the surface pattern is observed on the top view of many actual shoot apices [10, 14]. Notice the sector boundaries in the surface cell pattern of the spruce shoot apex shown in Fig. 10a. The angular variation of the cell walls that form the zigzags (Fig. 10b) is similar to those coming from the simulations in Fig. 6a where not uniform initials and the cell divisions including the random factor (mode II) were assumed (for the Kruskal–Wallis test we obtained H(2, N = 147) = 0.042 p = 0.98). In Fig. 10c three cell tetrads coming from the surface cell pattern of the Arabidopsis shoot apex adopted from Willis et al. [42]. On the basis of the temporal sequence coming from in vivo studies published in the quoted paper it was possible to suppose that their common point may correspond to the dome tip. If there was so, in cell pattern of each tetrad two initial divisions were manifested. Under such assumption angular variation of initial divisions was determined. The same was done with the cell tetrads coming from virtual shoot apex taking data from t_9_ in Fig. 7a as the example. The results are shown in Fig. 10d. It is easy to note that concerning preferred orientation of division walls there are large similarities between both angular distributions. Moreover, there is no significant differences between the angular distribution obtained for Arabidopsis apex and the distribution of all initial divisions observed during the whole considered simulation (see Additional file 7: Figure S2f). For Kruskal–Wallis test we obtained H (2, N = 52) = 4.28 p = 0.12.

## Discussion

A method to generate the surface cell layer of the paraboloidal shoot apical dome was presented. The surface layer originated from three apical initials and was developed through growth and cell divisions under isotropy of the surface growth. The cells, which were described by polyhedrons, divided anticlinally with the smallest division plane into two more or less equal daughters. The simulations showed how the cell pattern of the surface cell layer is formed and then develops step by step at successive times. A useful tool to study different aspects of the surface layer has been demonstrated. The obtained results and possible further application of the method are discussed in three areas: (1) apical initials and the surface cell pattern, (2) anticlinal cell divisions (3) further modeling.

### Apical initials and the surface cell pattern

The present method shows for the first time how the surface cell layer of the shoot apical dome is formed from the apical initials. The surface layer included about 350 cells in the region limited to the apical dome. Its formation required about 1200 cell divisions of both the apical initials and their descendants. Because these divisions were distributed more or less equally between three clonal sectors there were about 400 divisions per sector. The simulations showed that each apical initial renewed itself 7–8 times to generate the entire sector through the further divisions of their derivatives. A similar activity of initial cells was observed in empirical studies. Puławska [43] investigated the cellular clones that were recognized in the surface cell pattern of the *Actinidia arguta* shoot apex. Based on a clonal analysis, she concluded that the clones derived from four initials and that 4–8 divisions were needed to produce the entire surface layer. The L1 layer of *Arabidopsis* shoot apex includes about 200 cells in its dome-shaped part [35]. If they derive from three apical initials [44, 45], our results indicate that not more than 6–7 divisions per each initial are sufficient to produce this number of cells. Willis and other [42] observed two divisions during 80 h in the cells located near the dome tip. Similar activity of apical initials is suggested by Burian et al. [5] on the basis of studies of the fates of cells using in vivo laser ablation experiments and quantitative cell-lineage analysis. They found that usually 5–7 divisions per initial occur to displace the derivatives of 3–4 initial stem cells from the population of the apical meristem to the axial meristem, which has progenitor cells near the dome base.

Three clonal sectors with dynamically changing boundaries that met at the dome summit were formed in the surface cell pattern. The sectors were relatively stable and symmetrical due to the permanent status of the apical initials. In order to form such sectors, the cellular center in which the apical initials meet must coincide with the dome summit, i.e. the geometric central point of the dome surface [9, 10]. In our model this was achieved by two assumptions. Firstly, the GT field operated steadily so that its growth surface, which was responsible for surface growth, really corresponded to the dome surface (both represented by *v*_*s*_) and both symmetry axes of the field and the dome overlapped. Secondly, the common edge of the three apical initials was situated along the dome axis and its proximal end was situated at the dome summit. The three clonal boundaries that meet at the geometric center of the apical dome have been recognized in two microphotographs shown in this paper (Figs. 1a, 10a). A triad-type surface cellular pattern was observed in other studies of *Picea abies* and *Magnolia* shoot apices [10]. Its occurrence also results from the epoxy replicas studies of *Anagallis* [8], and may result from the time-laps confocal stacks presented recently by Willis and other [42] for *Arabidopsis*.

Two triads of cells were assumed to represent the apical initials at the beginning of the simulation. The uniform initials (In1), which were relatively large and equal in size, reached the critical volume and divided together almost at once, whereas the not uniform initials (In2), which were different, divided later and successively. During further development from both In1 and In2 similar sectors concerning their area were obtained when the surface layer was entire, and looking at their cell wall arrangement it is impossible to say whether they were originated from the uniform or not uniform initials. Obviously, in real shoot apices the occurrence of not uniform initials is more probable, but the uniform ones, as those manifested themselves the surface cell pattern of the young spruce seedling (Fig. 1b) can also happen. Notice that three cellular clones observed in this cell pattern are so equal and symmetric like those obtained by computer (t_3_ in Fig. 4), in our simulations derived from the uniform initials.

### Anticlinal cell divisions

The surface cell layer developed as a result of the growth and anticlinal cell divisions. The growth was defined by the field growth rates of a tensor type, whereas the cell divisions were created with the smallest division plane (SAD), defined geometrically. Because in the tensor field of growth rates there are the principal growth directions (PDGs) which are postulated to affect an orientation of cell divisions [23, 32, 46], the question arises how the divisions oriented according to the SAD rule relate to PDGs. The PDGs are three mutually orthogonal directions along which R_l_ attains extreme values: maximal, minimal and an intermediate one of a saddle type. They are determined at each point of GT field. Hejnowicz [32, 46] postulated that cells divide with respect to PDGs is such a way that a division wall lies typically in the plane defined by two PDGs at the site of its formation, which means that it is perpendicular to the third PDG. If the growth is locally anisotropic, the PDGs are easy to recognize. Each of the three PDGs can define cell division [46], but the division plane that is perpendicular to the direction along which R_l_ reaches the maximum is the most probable, if the differences in the values of R_l_ between particular PDGs are taken into account. When growth is locally isotropic in contrast, there are no distinguishable directions and each of them can represent PDG. Assuming that two directions that define the smallest division plane represents PDGs, such the plane, postulated by the Errera [30], is also oriented properly also with respect to PDGs. The case of isotropic surface growth assumed for the apical dome is slightly different in the sense that values of R_l_ are only the same in the plane tangent to the surface. Let us look at the shape of the 3D indicatrices for points of the dome surface (Fig. 2a). It is easy to note that the PDG along which R_l_ reaches the minimum always corresponds to ***a***, whereas two remaining PDGs must lie in the plane tangent to the surface. However, in this plane there are no differences in the values of R_l_ between particular directions and moreover, the R_l_ reaches the same maximal value along all of them. Therefore, each two mutually orthogonal directions can represent the remaining PDGs. To select them the criterion of the smallest division plane was used, but only in the application to the planes oriented anticlinally passing either through the cell centre in mode I, or the point found randomly near this centre in mode II. Accordingly, the division plane created by the SAD rule lies properly also with respect to PDGs. Moreover, such anticlinal division is evidently more probable than the periclinal one (one perpendicular to ***a***) due to small value of R_l_ along ***a***. This may explain why periclinal divisions are observed very seldom in the L1 cell layer.

From the point of view of mechanics growth is a kind of an irreversible strain of the cell wall system [24]. Because of turgor pressure the wall is under a tensile stress, usually anisotropic, but apart from this there are also tissue stresses [47], depending on an overall plant body structure and geometry. Therefore, the strain resulting from particular distribution of growth rate is a function of tensile stress of the cell walls. The stress, similarly to the growth rate, is the second-rank tensor quantity which defines its principal stress direction (PDS). Moreover, the stress and growth rate (strain) tensors are related to one another so that the directional cues included in PDGs may be related to the stress. How the stress influences cell divisions is still open question. A remarkable evidence that plant cells recognize the PDS comes from empirical studies [18, 33, 48]. According to Alim et al. [34] cell divisions following the shortest new wall reduce growth heterogeneity by actively enhancing the regulation of growth by mechanical stresses. On the basis of similarities between the pattern of PDS trajectories recognized in Lynch and Lintilhac [33] experiment and the pattern of PDG trajectories in the root apex Nakielski [49] suggested that proliferative divisions in the root proper, perpendicular to the PDG of the maximal R_l_, are tangent to the lines of maximal compression stress. In the shoot apex the tensile stress in the outer cell wall of the surface layer is predicted to be isotropic in the region surrounding the tip, whereas at the meristem flanks, the stress changes into anisotropic with maximal tensile stress in the circumferential direction [18, 50]. The shoot apex modelled in this paper was limited for the dome-shaped part so that there should be isotropic distribution of the tensile stress. Moreover, because the organ surface is the principal surface of the stress, defined by trajectories of two PDS tangent to the surface, the third PDG must be perpendicular to the surface. The used GT field was such that in each direction tangent to the surface R_l_ reached maximum, whereas R_l_ in the direction perpendicular to the surface was the smallest. In the plant cell the highest growth rate occurs usually where the tensile stress is the lowest [24]. Therefore, we have a good basis to suppose that each division wall created by SAD rule was defined by two principal directions: one corresponding to maximal R_l_ and minimal stress, and the other corresponding to minimal R_l_ and maximal stress. How mechanical properties of cell walls such as texture, chemical composition, plasticizing factors like expansions modulated by turgor pressure are regulated genetically is an open question. A link between genetic regulation and the definition of form and size during morphogenesis is a subject of advanced modeling studies in which various mechanical signals are taken into account [18, 51–54]. In general there is a continuous feedback between the actual form and the stress tensor, according to which the stress field in the walls of cells under turgor pressure and tissue stress depends upon actual cell shape and actual tissue organization of the organ. Due to such feedback the results even limited to the development of the cell wall system of the organ growing steadily give some information about possible distribution of the stress.

In the algorithm that was used, two modes relating to the position of the division plane with respect to the geometrical cell center were tested. In mode I, the plane passed precisely through the center, while in mode II it passed through a point that was randomly found within a small spherical region surrounding this center. Assuming the mode I, each simulation resulted in the same surface cell pattern, which only depended on the triad of apical initials that were assumed at the input (Fig. 5a, b). In mode II, a slightly different surface cell pattern was obtained in each simulation (Figs. 6, 7) due to presence of the random factor. Shoot apices from the same species usually manifest a similar cellular organization but differ from each other in the details of the cell wall arrangement. Therefore, mode II gave more realistic results than mode I, especially that the assumed randomness may be interpreted as being related to the position of the nucleus within the cell, which is not always central [55]. It is also important that the spherical region was small so that both of the daughter cells that were obtained through cell division had statistically similar volumes (Fig. 9). Moreover, the random factor influenced orientation of cell division walls only slightly, and resulted in a more or less equal division (how dimension of the spherical region affects volumes of the daughter cells—see Additional file 8: Figure S3). In this way the division plate halved the cell without an additional assumption which is primarily used to specify the position of the division plate in Errera’s rule [35, 36, 38]. A similar way of modifying the division plane that was defined for other division rules was recently tested using the 2D approach [49, 56, 57]. The examples of much stronger changes in the position and orientation of the division plane that were influenced by a random factor were described by Sahlin and Jonson [38] and Alim et al. [34].

The present model allowed us to study cell divisions in two aspects—their spatial distribution and angular variation. During the whole simulation about 1700 divisions that originated from uniform initials and 1550 from not uniform initials were observed. All of the divisions that were considered together in the top view were distributed uniformly through the surface (Fig. 8a, d); whereas a similar distribution that was limited only to the apical initials indicated that the division walls occupy three specific areas that are situated more or less at the same distance from the dome summit. Interestingly, not only their location but also their arrangement corresponded to the first approximation with the apical initials that was assumed at the input (Fig. 8b, e). To compare the number of cell divisions between particular simulations in more detail, the same time-period was used. This period corresponded to the 2-h intervals that are usually used in empirical studies of cell divisions in the *Arabidopsis* shoot apex [35]. We observed 9–11 divisions with a random and quantitatively equal number distribution between the sectors in mode I (Fig. 5a, b) and 10–25 divisions with a random and mostly unequal distribution between the sectors in mode II (as 8:1:6 and 2:7:3 in Fig. 6b). These results, especially in mode II, are consistent with the microscopic data published by Shapiro et al. [35]. Interestingly, in both our simulations and in actual apices, there are examples in which sometimes a surprisingly large or small number of divisions occur.

In regards to the angular variations in the division walls, we concluded that the divisions were distributed uniformly in for mode I (Fig. 5a, b) and less uniformly but still without statistically significant differences in mode II (Figs. 6, 7). For the case in which the uniform initials (In1) and mode II were used, small maxima (Fig. 6) were also observed in the distribution functions (Fig. 9b). However, they were too small to indicate some preferences in the orientation of the division walls statistically. More significant maxima appeared in the angular distributions in which the divisions of the initial cells were considered individually in both mode I (Fig. 8c, f) and mode II (Additional file 7: Figure 2). If these maxima indicate some directional preferences in the orientation of the divisions of the apical initials, the question arises where do they come from? Figure 8b, e suggest that they may be linked with the shape of the apical initials assumed at the input, in particular the orientation of the three-way junction between the anticlinal walls at the dome summit.

### Future modeling

Superficial layer of cells is the object of many empirical studies in vivo, performed by advanced confocal microscopy, in particular on the Arabidopsis shoot apex. These studies, focused on the problem how the cell expansion and cell divisions are coordinated and regulated during growth [5, 35, 42], receive useful information about development of the surface cell pattern. Such the cell pattern, after limitation to the region of the apical dome could be introduced as incoming data to our model and used to simulate growth during the time period much longer than 2–3 days commonly achieved experimentally. In our simulations time-period is practically unlimited, in this paper it was at least four times longer than in the case of the longest time period demonstrated in time-lapse confocal stacks published by Willis et al. [42]. We do hope that our computer modeling complements empirical studies in the area which is difficult for direct experimental exploration.

The configuration in which the three apical initials meet at the dome summit is geometrically stable, especially under steady isotropic surface growth. However, it is reasonable to suppose that in actual shoot apices, the cellular center does not usually coincide with the summit. A clonal analysis of the surface cell pattern showed [10] that the cellular center, where the clonal boundaries of the three sectors meet, can be shifted towards the side of the meristem. The shift, which is interpreted as a tilting of the meristem axis, triggers reorganization between the cells of the apex and finally leads to another configuration in which 1 or 4 cells become the apical initials. Based on such observations, Zagórska-Marek and Turzańska [10] concluded that the position and number of initial cells may change over time. The ability of meristems to change initials themselves is clear but a change in their number needs further studies. For example, in no bifurcating *Huperzia* shoots typical tetrad of initial cells may function temporality and be replaced by new ones, but the same configuration (still a tetrad of cells) persists [58]. Also our preliminary simulations in which a triad of initials was assumed indicates that there could be again three apical initials, now at new position, in such a case. The large variety of cell tetrads that were observed in very apical region (t_9_ in Figs. 5, 6, 7) seems to support such view. Even under three permanent initials, we had doubts about which of the four cells that were located in this region was the apical initials and where the cellular center was located. Only by taking into account the growth trajectories that always run meridional from the dome summit as well as the corresponding cell arrangements that were visible from the side view, were we able to give clear answers to these questions.

The derivative sector was composed of its apical initial, the initial sister and a number of cellular clones that had been initiated by the previous self-renewing initials in the same initial lineage. When the surface layer was complete (t_8_-top view in Fig. 4), each sector included 6–7 cellular clones of different ages. These clones increased in cell number through the subsequent divisions of their cells and were successively displaced further away from the dome summit during growth. Analysis of their cell pattern from the top view showed that the subsequent self-renewing divisions of the apical initial are manifested in the shape, alignment and orientation of the particular clones. Taking all of the clones into account, it was possible to determine the history of the cell divisions in a given sector. Having data from all of the sectors, the subsequent stages of cellular organization could be studied. In this respect, the clonal analysis of our virtual cell layer offers information that is similar to that from microscopic observations of real shoot apices [8, 59], but coming from a much longer time interval, which is usually not feasible in experimental studies.

Some of the problems that are related to the proposed method of the formation of the surface cell layer of the shoot apex are open for further research. In this paper the method was implemented on the example of the apical dome with a paraboloidal shape, well described in terms of GT field. However, it can be applied to domes that have a different shape as well as the surface growth other than isotropic. The examples of GT field dedicated for elliptically and hyperbolically shaped apices that grow under the anisotropic surface growth were described [26]. In order to be as close as possible to empirical data the case in which in the lateral region near the dome base the isotropic surface growth changes into anisotropic would be interesting, especially that the method how to define GT filed composed of the zones that differ each other concerning a type of growth under continuity of the displacement velocity field can be adopted from previous applications to root apices [13, 40, 60].

Next problem is whether the GT field must be steady. In this paper, it was steady to have the initials permanent. However, the same model allows unsteady growth which is generated by changing the operational application of the growth field to the meshwork that represents the surface cell pattern. The examples of the previous modeling have shown [61, 62] that we are able to redefine directional information about PDGs received by the cells at their hitherto positions. In application to our model the cases of the impermanence of the apical initials [10] can be simulated in this way.

Cell proliferation is an important factor that affects the cellular geometry of a growing tissue [55, 63]. The simulation model, which was used in this study to generate a single virtual surface cell layer of the shoot apex, offers a unique tool to investigate how cell divisions interpreted in terms of the tensor–based approach affect the development of cell pattern of an organ when it is considered as a 3D structure. The view that three apical initials that form three vegetative clones occur in the shoot apex has been known for years, but only now, by using the model we were able to observe how that is done. The other view also Korn [9, 64] postulated the occurrence of a single apical initial that is located at the dome summit. With one apical initial the sectors also arise, but through a more complex growth pattern in which the apical initial occasionally divides periclinally to form a new apical initial of the tunica and the sister cell of the underlying corpus. This idea is interesting, especially that the occurrence of the single initial is suggested to result from the fastest growth in the very apical region of the dome which really exists in the GT field that we used (Fig. 2a), but its verification goes beyond the framework of the present model.

## Conclusions

The present method based on the growth tensor approach, is a useful tool to generate formation of the surface cell layer. It is convenient to study development of surface cell pattern much longer than in empirical studies, monitoring activity of initial cells and their derivatives. Different types of studies can be developed including those related to the problem how cell expansion and division are coordinated during growth. We expect its further application to clarify such question as the number of apical cells and whether these initials are permanent or not, and possible relationship between shape of apical initials and their oriented divisions during growth. In such studies the shoot apex coming from particular species can be modelled, assuming tensor field of growth rates other in the sense that based directly on empirical data adopted from in vivo studies.

## Additional files

**Additional file 1.** Calculation of growth rates for the paraboloidal shoot apical dome with isotropic surface growth.

**Additional file 2: Figure S1.** The block diagram showing the iteration method used in the model.

**Additional file 3: Video S1.** Formation of the surface cell layer that derives from **uniform** apical initials (In1) and develops with cell divisions in **mode I** (side and top views).

**Additional file 4: Video S2.** Formation of the surface cell layer that derives from **not uniform** apical initials (In2) and develops with cell divisions in **mode I** (side and top views).

**Additional file 5: Video S3.** Formation of the surface cell layer obtained for three apical domes that derive from **uniform** initials (In1) and develops with cell divisions in **mode II** (top views, for one of the dome in magnification).

**Additional file 6: Video S4.** Formation of the surface cell layer obtained for three apical domes that derive from **not uniform** apical initials (In2) and develops with cell divisions in **mode II** (top views, for one of the dome in magnification).

**Additional file 7: Figure S2.** Angular orientation of division walls obtained for all cells (left) and the apical initials only (right) in the simulations that assumed: (a) uniform initials and cell divisions in mode I, data from the simulation in Fig. 5a; (b–d) uniform initials and cell divisions in mode II, data from the simulation in Fig. 6a–c; (e) initials In2 and cell divisions in mode II, data from the simulation in Fig. 5b; (f–h) initials In2 and cell divisions in mode II, data from the simulation in Fig. 7a–c.

**Additional file 8: Figure S3.** Gaussian approximation applied to distribution of the daughter cells volume obtained in four simulations in which different the circular regions deteriming localization of division wall within the cells were assumed. The following values of the radius were considered: *ρ* = 0 (black), *ρ* = 0.002 (green), *ρ* = 0.02 (red), *ρ* = 0.2 (blue). The value 1.0 on the horizontal axis means exactly the same volume of both daughters.

## Abbreviations

GT: growth tensor
PDG: principal direction of growth
PDS: principal direction of stress
SAD: smallest anticlinal division
