# Supplementary material for: A method to generate the surface cell layer of the 3D virtual shoot apex from apical initials
Source: Plant Methods. 2017 Dec 11;13:110. doi: 10.1186/s13007-017-0262-7 (PMC5725887; doi:10.1186/s13007-017-0262-7)
Supplement: Supplementary file 1 — Additional file 1. Calculation of growth rates for the paraboloidal shoot apical dome with isotropic surface growth. [file 13007_2017_262_MOESM1_ESM.pdf]

Calculation of growth rates for the paraboloidal shoot apical dome with isotropic surface growth

The growing shoot apex is characterized by the continuous field of the displacement velocity **V**. Knowing **V**, the relative elemental rate of growth in line (R_l) which is a measure of the growth at a point can be determined. This quantity may attain different values at a point depending on a direction. For a particular direction, $\mathbf{e}_s$,the R_l can be obtained directly from the definition (Hejnowicz and Romberger 1984, Hejnowicz 1989):

$$R_{l(s)} = \lim_{\substack{\Delta t \to 0 \\ \Delta s \to 0}} \frac{\Delta(\Delta s)}{\Delta s \Delta t} = \frac{d\frac{ds}{dt}}{ds} = \frac{dV_s}{ds} = \frac{d(\mathbf{V} \cdot \mathbf{e}_s)}{ds} = (\nabla\mathbf{V}) \cdot \mathbf{e}_s \cdot \mathbf{e}_s$$

where $(\nabla\mathbf{V})$ is a dyadic (gradient taken from vector), $\mathbf{e}_s$ is a unit vector of the direction, and each dot means the scalar product. The dyadic multiplied twice by $\mathbf{e}_s$ in the coordinate system having p, q =1, 2, 3 as unit vectors, is equivalent to $T_{pq}e^p e_q$ where, using summation convention, T_{pq} represents a second rank operator called the growth tensor, GT (Hejnowicz and Romberger 1984). The GT defines the field of growth rates of the organ. If data about **V** come from empirical studies, the growth field obtained in this way can be regarded as representative for the considered organ (Hejnowicz et al. 1989). As this field is of a tensor type, locally unless growth is isotropic there are three mutually orthogonal principal growth directions, PDGs, along which the R_l attains extreme values (maximal, minimal and intermediate one of a saddle type). The GT can be calculated in any coordinate system, however, using the system called natural (Hejnowicz 1984) in which unit vectors are postulated to represent PDGs at the organ level, it attains the simplest form.

For the paraboloidal shaped shoot apex, the paraboloidal coordinate system (u, v, φ) was proposed as the natural one (Hejnowicz et at. 1984). This system, shown below for φ=0, is curvilinear, orthogonal and of a confocal type. It has a rotational symmetry, with the lines u=0 and v=0 as the symmetry axis. For the unit vectors $\mathbf{e}_u$, $\mathbf{e}_v$, $\mathbf{e}_\varphi$ representing PDGs, through every point pass three coordinate lines corresponding to PDG trajectories: periclinal, anticlinal and latitudinal (the latter is perpendicular to the figure plane). Pairs of these trajectories define the principal surfaces, in 3D described by u=const., v=const., and φ=const.; one of these surfaces, namely $v=v_s$ corresponds to the shoot apex surface.

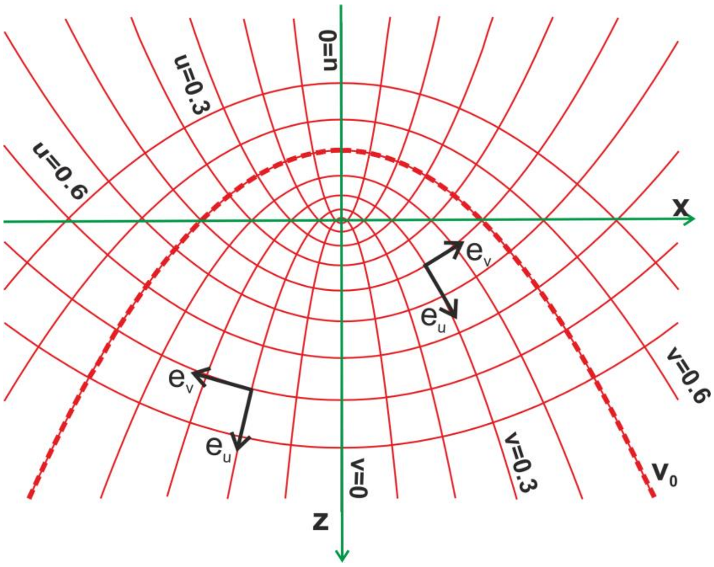

The equations defining the paraboloidal system are the following:

$$x = uv\cos(\varphi), \quad y = uv\sin(\varphi), \quad z = ½\,(u^2 - v^2)$$

$$u = \sqrt{\sqrt{x^2+y^2+z^2}+z} \qquad v = \sqrt{\sqrt{x^2+y^2+z^2}-z} \qquad \varphi = arctg\,\frac{y}{x}$$

In the paraboloidal system, **V** vector has three physical components: $V_u = h_u\frac{\partial u}{\partial t}$ $\quad V_v = h_v\frac{\partial v}{\partial t}$ $\quad V_\varphi = h_\varphi\frac{\partial \varphi}{\partial t}$

where $h_u = h_v = \sqrt{u^2+v^2},\ h_\varphi = uv$ are scale factors of the system, whereas $\frac{\partial u}{\partial t}, \frac{\partial v}{\partial t}, \frac{\partial \varphi}{\partial t}$ define **V** in the curvilinear coordinates.

The GT in any coordinate system with a rotational symmetry is given by the matrix (Nakielski 1987):

$$GT = \begin{bmatrix} T_{uu} & T_{uv} & T_{u\varphi} \\ T_{vu} & T_{vv} & T_{v\varphi} \\ T_{\varphi u} & T_{\varphi v} & T_{\varphi\varphi} \end{bmatrix},$$

where

$$T_{uu} = \frac{1}{h_u}\left(\frac{\partial}{\partial u}\frac{\partial u}{\partial t} + \frac{1}{h_v}\frac{\partial h_u}{\partial v}\frac{\partial v}{\partial t}\right) \quad T_{uv} = \frac{1}{h_u}\left(\frac{\partial}{\partial u}\frac{\partial v}{\partial t} - \frac{1}{h_v}\frac{\partial h_u}{\partial v}\frac{\partial u}{\partial t}\right) \quad T_{u\varphi} = \frac{1}{h_u}\frac{\partial}{\partial u}\frac{\partial \varphi}{\partial t}$$

$$T_{vu} = \frac{1}{h_v}\left(\frac{\partial}{\partial v}\frac{\partial u}{\partial t} - \frac{1}{h_u}\frac{\partial h_v}{\partial u}\frac{\partial v}{\partial t}\right) \quad T_{vv} = \frac{1}{h_v}\left(\frac{\partial}{\partial v}\frac{\partial v}{\partial t} + \frac{1}{h_u}\frac{\partial h_v}{\partial u}\frac{\partial u}{\partial t}\right) \quad T_{v\varphi} = \frac{1}{h_v}\frac{\partial}{\partial v}\frac{\partial \varphi}{\partial t}$$

$$T_{\varphi u} = \frac{1}{h_\varphi}\left(\frac{\partial}{\partial \varphi}\frac{\partial u}{\partial t} - \frac{1}{h_u}\frac{\partial h_\varphi}{\partial u}\frac{\partial v}{\partial \varphi}\right) \quad T_{\varphi v} = \frac{1}{h_\varphi}\left(\frac{\partial}{\partial \varphi}\frac{\partial v}{\partial t} - \frac{1}{h_v}\frac{\partial h_\varphi}{\partial v}\frac{\partial \varphi}{\partial t}\right) \quad T_{\varphi\varphi} = \frac{1}{h_\varphi}\left(\frac{\partial}{\partial \varphi}\frac{\partial \varphi}{\partial t} + \frac{1}{h_u}\frac{\partial h_\varphi}{\partial u}\frac{\partial u}{\partial t} + \frac{1}{h_v}\frac{\partial h_\varphi}{\partial v}\frac{\partial v}{\partial t}\right),$$

In the above GT matrix all components of **V** are represented, and T_{uu}, T_{vv}, $T_{\varphi\varphi}$ indicate values of R_l in $\mathbf{e}_u$, $\mathbf{e}_v$, $\mathbf{e}_\varphi$, respectively. In the present work, we are interested in the shoot apex growing steadily and without a rotation around its symmetry axis, which means that $\frac{\partial v}{\partial t} = 0$, $\frac{\partial \varphi}{\partial t} = 0$. Moreover, growth at the surface of the apex has to be isotropic which means that for each point of the surface, R_l is the same in any direction, in the plane tangent to this surface. The dome surface is represented by $v=v_s$, whereas the plane tangent to the surface is defined by $\mathbf{e}_u$ and $\mathbf{e}_\varphi$. Accordingly, for every point at the surface there must be $T_{uu} = T_{\varphi\varphi}$. Specifying the scale factors for $v= v_s$ we obtain:

$$\frac{1}{\sqrt{u^2+{v_s}^2}}\frac{\partial}{\partial u}\frac{\partial u}{\partial t} = \frac{1}{u\sqrt{u^2+{v_s}^2}}\frac{\partial u}{\partial t}$$

Integration gives $\frac{\partial u}{\partial t} = cu$ only for points the dome surface; c is the integration constant. By means of the scaling factors we are able to extend this expression on points of the whole dome:

$$\frac{\partial u}{\partial t} = \frac{c\sqrt{u^2+v^2}}{\sqrt{u^2+{v_s}^2}}\, u$$

Introducing $\frac{\partial u}{\partial t}$ into the general form of the growth tensor we obtain the GT in specification appropriate to the considered shoot apex (Hejnowicz et al. 1984, Nakielski 1987):

$$GT = \frac{1}{(u^2+v^2)\sqrt{u^2+{v_s}^2}}\begin{vmatrix} \frac{u^4+2u^2{v_s}^2+v^2{v_s}^2}{u^2+{v_s}^2} & -uv & 0 \\ uv & u^2 & 0 \\ 0 & 0 & u^2+v^2 \end{vmatrix}$$

Having GT, the R_l in any direction $\mathbf{e}_s$ can be calculated according to the equation:

$$R_{l(e_s)} = [a_u, a_v, a_\varphi]GT\begin{bmatrix} a_u \\ a_v \\ a_\varphi \end{bmatrix}$$

where

$$a_u = \boldsymbol{e}_u \cdot \boldsymbol{a}$$
$$a_v = \boldsymbol{e}_v \cdot \boldsymbol{a}$$
$$a_u = \boldsymbol{e}_\varphi \cdot \boldsymbol{a}$$

The unit vectors

$$\boldsymbol{e}_u = \left[\frac{v\,cos\varphi}{\sqrt{u^2+v^2}}, \frac{v\,sin\varphi}{\sqrt{u^2+v^2}}, \frac{u}{\sqrt{u^2+v^2}}\right]$$
$$\boldsymbol{e}_v = \left[\frac{u\,cos\varphi}{\sqrt{u^2+v^2}}, \frac{u\,sin\varphi}{\sqrt{u^2+v^2}}, \frac{-v}{\sqrt{u^2+v^2}}\right]$$
$$\boldsymbol{e}_\varphi = [-sin\varphi, cos\varphi, 0]$$

and

$$\boldsymbol{a} = [cos\psi\ sin\theta, sin\psi\ sin\theta, cos\theta]$$

where ψ and θ are the spherical coordinate of the $\boldsymbol{e}_s$ vector (azimuth and the height).
