# Supplementary figures and images for: A method to generate the surface cell layer of the 3D virtual shoot apex from apical initials
Source: Plant Methods. 2017 Dec 11;13:110. doi: 10.1186/s13007-017-0262-7 (PMC5725887; doi:10.1186/s13007-017-0262-7)

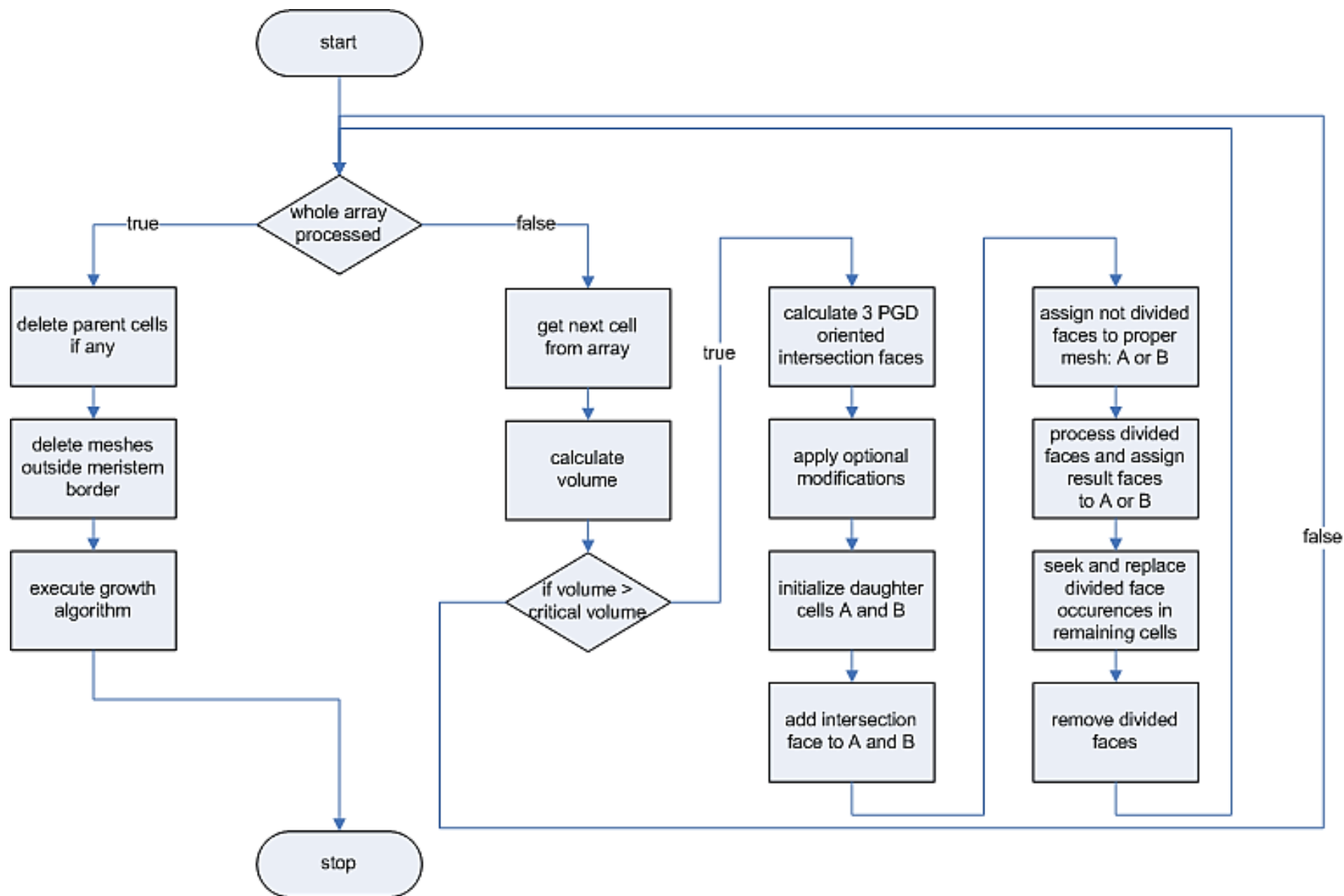

Supplement: Supplementary file 2 — Additional file 2: Figure S1. The block diagram showing the iteration method used in the model. [file 13007_2017_262_MOESM2_ESM.pdf]

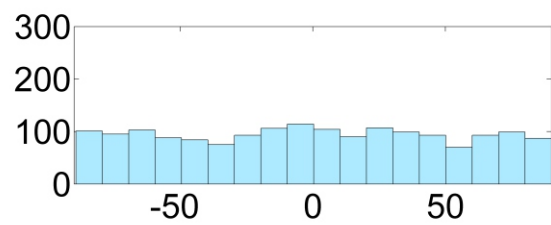

**a**

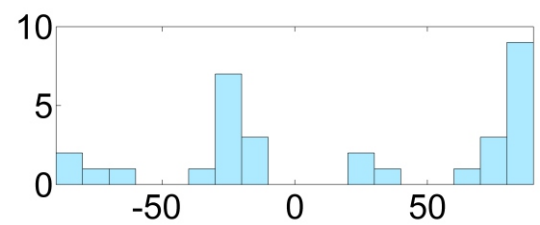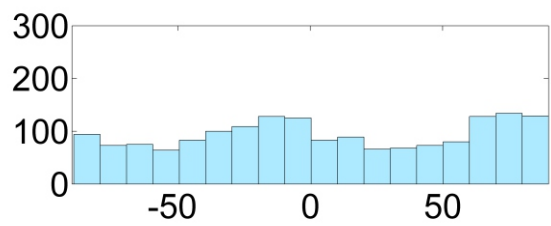**b**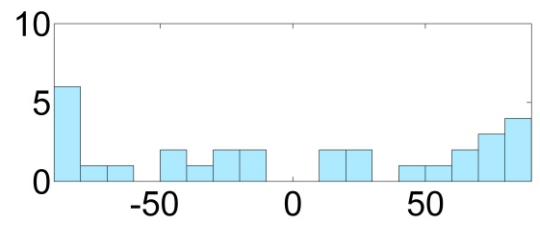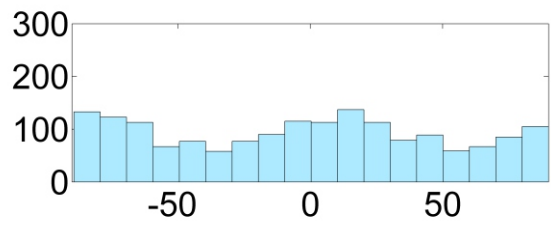

**C**

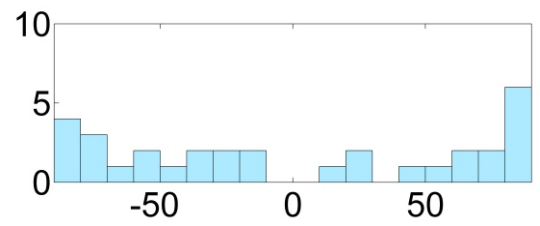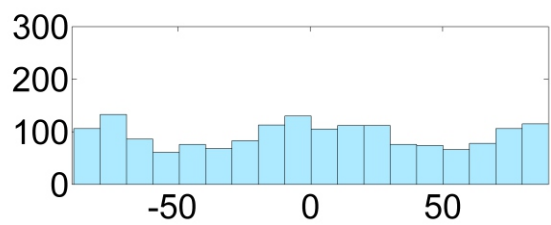

**d**

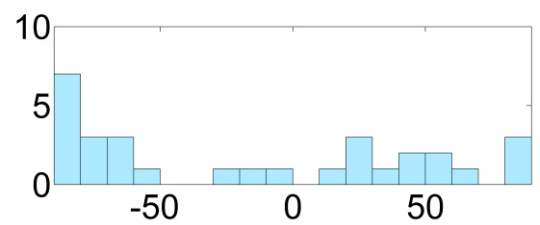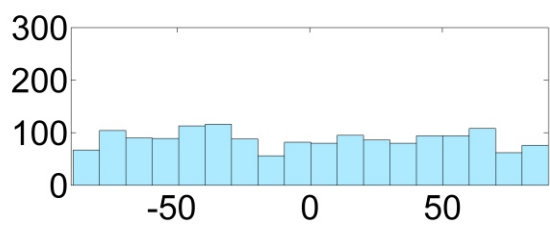

**e**

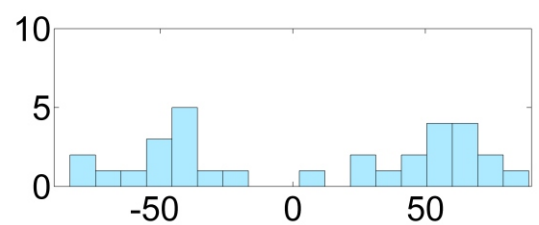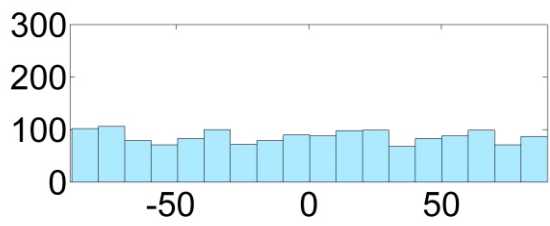**f**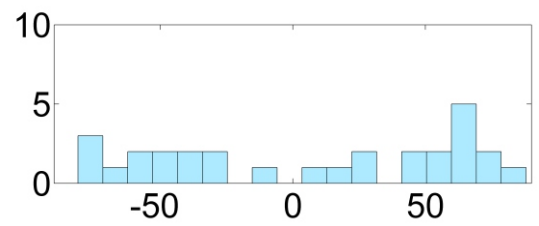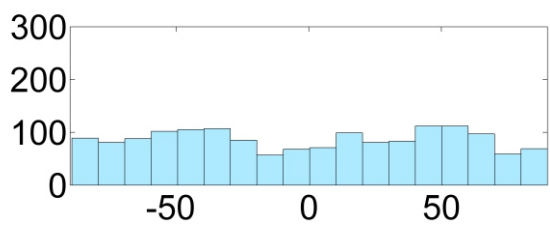

**g**

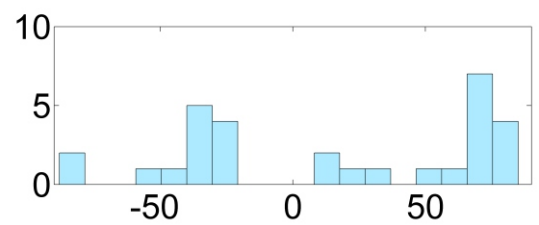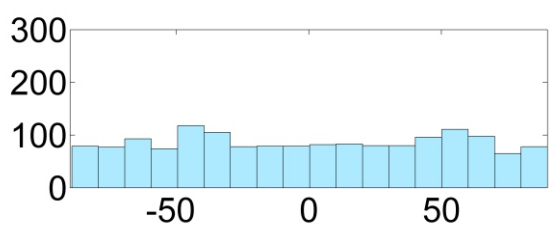

# h

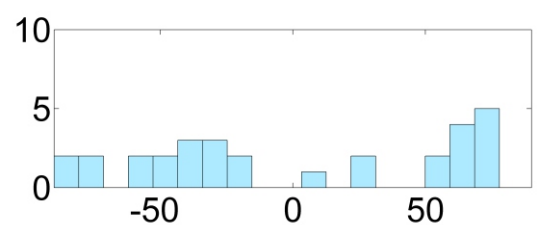

Supplement: Supplementary file 7 — Additional file 7: Figure S2. Angular orientation of division walls obtained for all cells (left) and the apical initials only (right) in the simulations that assumed: (a) uniform initials and cell divisions in mode I, data from the simulation in Fig. 5a; (b–d) uniform initials and cell divisions in mode II, data from the simulation in Fig. 6a–c; (e) initials In2 and cell divisions in mode II, data from the simulation in Fig. 5b; (f–h) initials In2 and cell divisions in mode II, data from the simulation in Fig. 7a–c. [file 13007_2017_262_MOESM7_ESM.pdf]

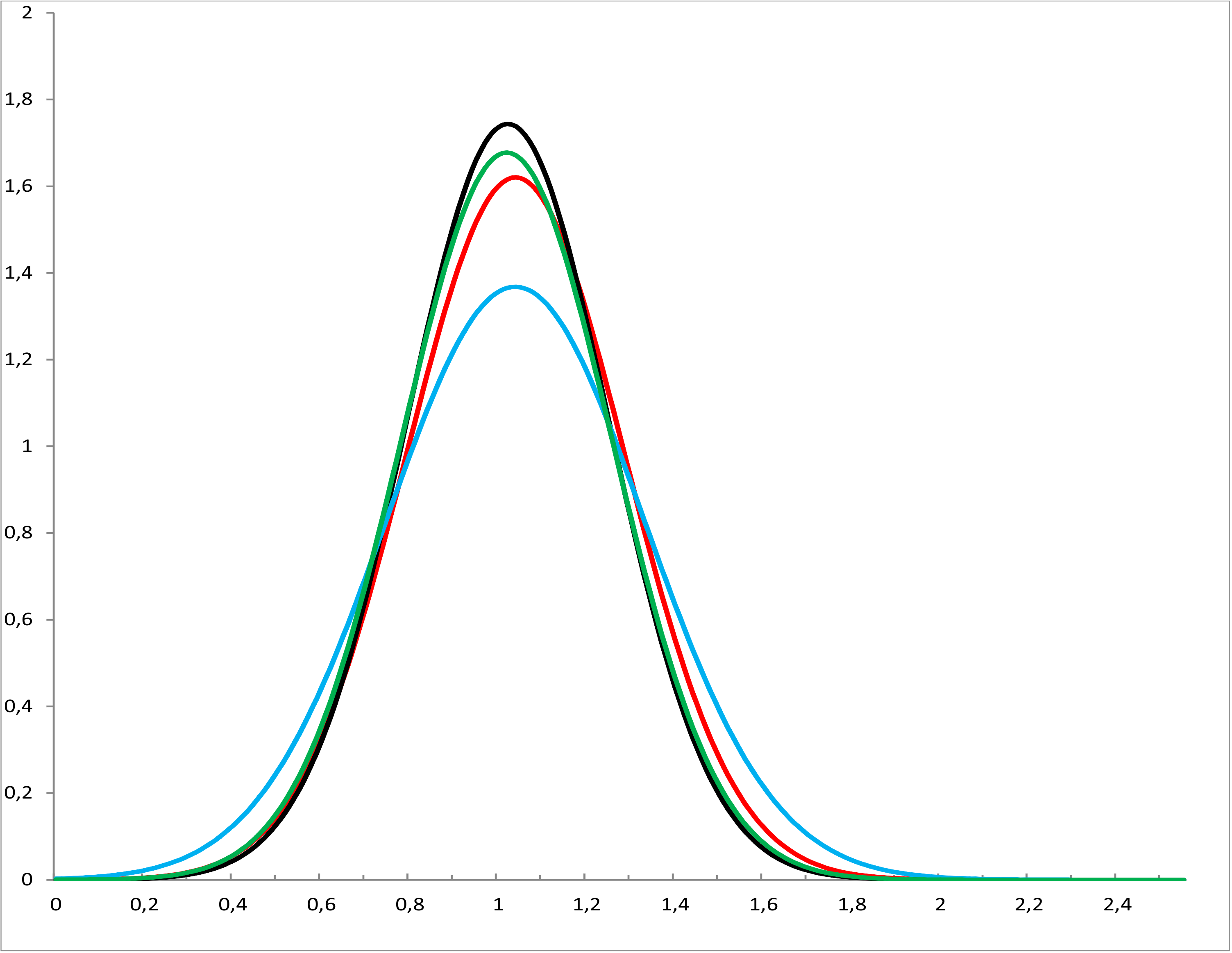

Supplement: Supplementary file 8 — Additional file 8: Figure S3. Gaussian approximation applied to distribution of the daughter cells volume obtained in four simulations in which different the circular regions deteriming localization of division wall within the cells were assumed. The following values of the radius were considered: ρ = 0 (black), ρ = 0.002 (green), ρ = 0.02 (red), ρ = 0.2 (blue). The value 1.0 on the horizontal axis means exactly the same volume of both daughters. [file 13007_2017_262_MOESM8_ESM.tif]
